# Supplementary material for: A truncated variant of the ribosome-associated trigger factor specifically contributes to plant chloroplast ribosome biogenesis
Source: Nat Commun. 2025 Jan 13;16:629. doi: 10.1038/s41467-025-55813-1 (PMC11731035; doi:10.1038/s41467-025-55813-1)
Supplement: Supplementary file 1 — Supplementary Information [file 41467_2025_55813_MOESM1_ESM.pdf]

Supplementary Information

A truncated variant of the ribosome-associated trigger factor specifically contributes to plant chloroplast ribosome biogenesis

Fabian Ries[1,2], Jasmin Gorlt[1], Sabrina Kaiser[3], Vanessa Scherer[4], Charlotte Seydel[5], Sandra Nguyen[1], Andreas Klingl[5], Julia Legen[6], Christian Schmitz-Linneweber[6], Hinrik Plaggenborg[9], Jediael Z. Y. Ng[7], Dennis Wiens[7], Georg K. A. Hochberg[7,8], Markus Räschle[9], Torsten Möhlmann[4], David Scheuring[3], and Felix Willmund[1,10]*

Author's institution(s)/affiliation(s):

[1] Molecular Genetics of Eukaryotes, University of Kaiserslautern, Paul-Ehrlich-Str. 23, 67663 Kaiserslautern, Germany.

[2] Present address: Institute of Systems Biotechnology, Saarland University, Campus A 1.5, 66123 Saarbrücken

[3] Plant Pathology, University of Kaiserslautern, Paul-Ehrlich-Str. 22, 67663 Kaiserslautern, Germany.

[4] Plant Physiology, University of Kaiserslautern, Paul-Ehrlich-Str. 22, 67663 Kaiserslautern, Germany.

[5] Plant Development, Ludwig-Maximilians-University Munich, Großhadernerstr. 2-4 82152 Planegg-Martinsried, Germany

[6] Molecular Genetics, Humboldt-University of Berlin, Philipp-Str. 13, 10115 Berlin, Germany.

[7] Max-Planck-Institute for Terrestrial Microbiology, Karl-von-Frisch-Str. 10, 35043 Marburg, Germany

[8] Evolution Biology & Synmikro, University of Marburg, Hans-Meerwein-Str 4, 35032 Marburg, Germany.

[9] Molecular Genetics, University of Kaiserslautern, Paul-Ehrlich-Str. 24, 67663 Kaiserslautern, Germany.

[10] Present address: Molecular Plant Sciences & Synmikro, University of Marburg, Karl-von-Frisch-Str. 14, 35032 Marburg, Germany.

***Corresponding author:** Felix Willmund (willmund@staff.uni-marburg.de)

Supplementary Figures and Legends

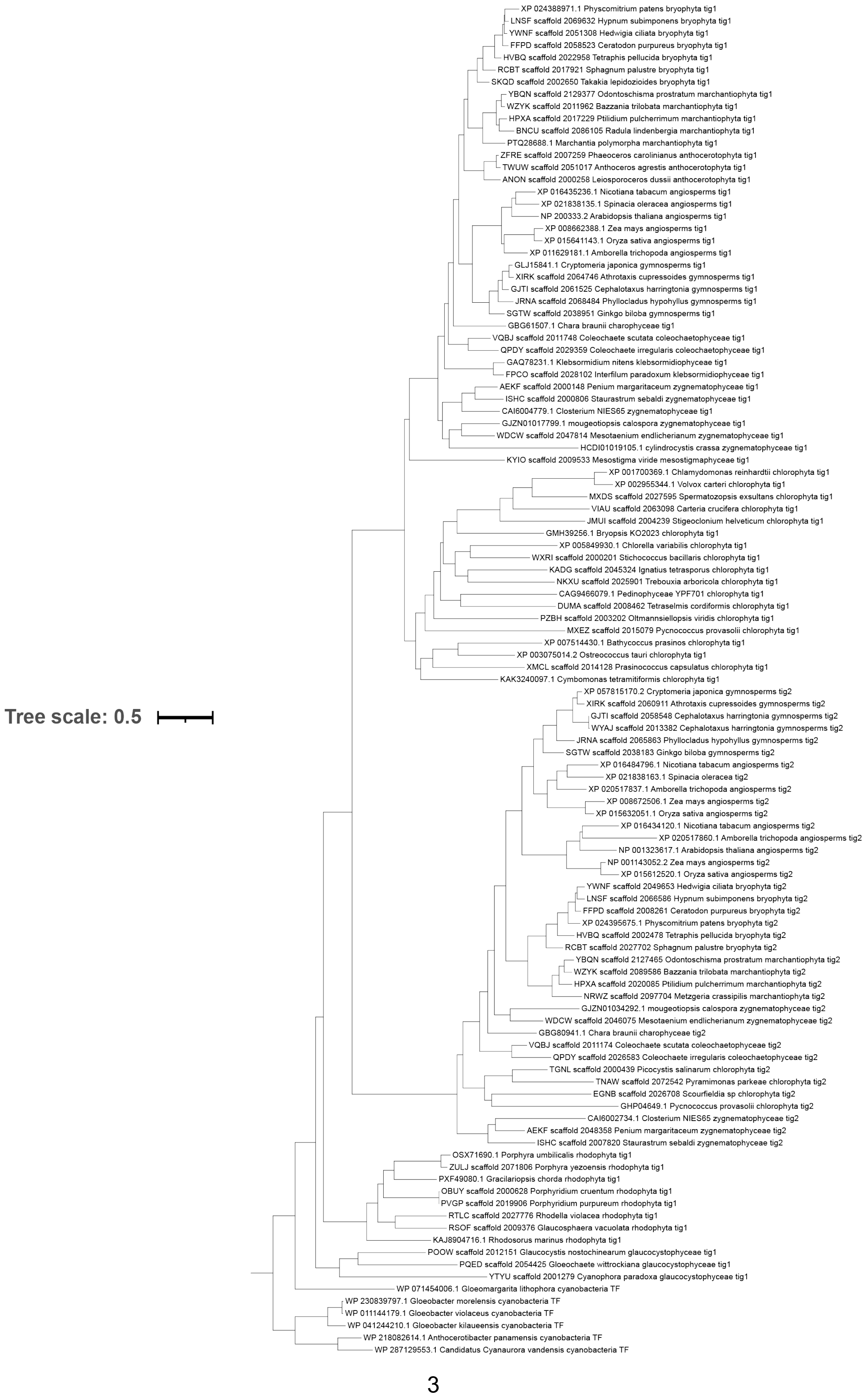
XP 024388971.1 Physcomitrium patens bryophyta tig1
LNSF scaffold 2069632 Hypnum subimponens bryophyta tig1
YWNF scaffold 2051308 Hedwigia ciliata bryophyta tig1
FFPD scaffold 2058523 Ceratodon purpureus bryophyta tig1
HVBQ scaffold 2022958 Tetraphis pellucida bryophyta tig1
RCBT scaffold 2017921 Sphagnum palustre bryophyta tig1
SKQD scaffold 2002650 Takakia lepidozioides bryophyta tig1
YBQN scaffold 2129377 Odontoschisma prostratum marchantiophyta tig1
WZYK scaffold 2011962 Bazzania trilobata marchantiophyta tig1
HPXA scaffold 2017229 Ptilidium pulcherrimum marchantiophyta tig1
BNCU scaffold 2086105 Radula lindenbergia marchantiophyta tig1
PTQ28688.1 Marchantia polymorpha marchantiophyta tig1
ZFRE scaffold 2007259 Phaeoceros carolinianus anthocerotophyta tig1
TWUW scaffold 2051017 Anthoceros agrestis anthocerotophyta tig1
ANON scaffold 2000258 Leiosporoceros dussii anthocerotophyta tig1
XP 016435236.1 Nicotiana tabacum angiosperms tig1
XP 021838135.1 Spinacia oleracea angiosperms tig1
NP 200333.2 Arabidopsis thaliana angiosperms tig1
XP 008662388.1 Zea mays angiosperms tig1
XP 015641143.1 Oryza sativa angiosperms tig1
XP 011629181.1 Amborella trichopoda angiosperms tig1
GLJ15841.1 Cryptomeria japonica gymnosperms tig1
XIRK scaffold 2064746 Athrotaxis cupressoides gymnosperms tig1
GJTI scaffold 2061525 Cephalotaxus harringtonia gymnosperms tig1
JRNA scaffold 2068484 Phyllocladus hypohyllus gymnosperms tig1
SGTW scaffold 2038951 Ginkgo biloba gymnosperms tig1
GBG61507.1 Chara braunii charophyceae tig1
VQBJ scaffold 2011748 Coleochaete scutata coleochaetophyceae tig1
QPDY scaffold 2029359 Coleochaete irregularis coleochaetophyceae tig1
GAQ78231.1 Klebsormidium nitens klebsormidiophyceae tig1
FPCO scaffold 2028102 Interfilum paradoxum klebsormidiophyceae tig1
AEKF scaffold 2000148 Penium margaritaceum zygnematophyceae tig1
ISHC scaffold 2000806 Staurastrum sebaldi zygnematophyceae tig1
CAI6004779.1 Closterium NIES65 zygnematophyceae tig1
GJZN01017799.1 mougeotiopsis calospora zygnematophyceae tig1
WDCW scaffold 2047814 Mesotaenium endlicherianum zygnematophyceae tig1
HCDI01019105.1 cylindrocystis crassa zygnematophyceae tig1
KYIO scaffold 2009533 Mesostigma viride mesostigmaphyceae tig1
XP 001700369.1 Chlamydomonas reinhardtii chlorophyta tig1
XP 002955344.1 Volvox carteri chlorophyta tig1
MXDS scaffold 2027595 Spermatozopsis exsultans chlorophyta tig1
VIAU scaffold 2063098 Carteria crucifera chlorophyta tig1
JMUI scaffold 2004239 Stigeoclonium helveticum chlorophyta tig1
GMH39256.1 Bryopsis KO2023 chlorophyta tig1
XP 005849930.1 Chlorella variabilis chlorophyta tig1
WXRI scaffold 2000201 Stichococcus bacillaris chlorophyta tig1
KADG scaffold 2045324 Ignatius tetrasporus chlorophyta tig1
NKXU scaffold 2025901 Trebouxia arboricola chlorophyta tig1
CAG9466079.1 Pedinophyceae YPF701 chlorophyta tig1
DUMA scaffold 2008462 Tetraselmis cordiformis chlorophyta tig1
PZBH scaffold 2003202 Oltmannsiellopsis viridis chlorophyta tig1
MXEZ scaffold 2015079 Pycnococcus provasolii chlorophyta tig1
XP 007514430.1 Bathycoccus prasinos chlorophyta tig1
XP 003075014.2 Ostreococcus tauri chlorophyta tig1
XMCL scaffold 2014128 Prasinococcus capsulatus chlorophyta tig1
KAK3240097.1 Cymbomonas tetramitiformis chlorophyta tig1
Tree scale: 0.5
XP 057815170.2 Cryptomeria japonica gymnosperms tig2
XIRK scaffold 2060911 Athrotaxis cupressoides gymnosperms tig2
GJTI scaffold 2058548 Cephalotaxus harringtonia gymnosperms tig2
WYAJ scaffold 2013382 Cephalotaxus harringtonia gymnosperms tig2
JRNA scaffold 2065863 Phyllocladus hypohyllus gymnosperms tig2
SGTW scaffold 2038183 Ginkgo biloba gymnosperms tig2
XP 016484796.1 Nicotiana tabacum angiosperms tig2
XP 021838163.1 Spinacia oleracea tig2
XP 020517837.1 Amborella trichopoda angiosperms tig2
XP 008672506.1 Zea mays angiosperms tig2
XP 015632051.1 Oryza sativa angiosperms tig2
XP 016434120.1 Nicotiana tabacum angiosperms tig2
XP 020517860.1 Amborella trichopoda angiosperms tig2
NP 001323617.1 Arabidopsis thaliana angiosperms tig2
NP 001143052.2 Zea mays angiosperms tig2
XP 015612520.1 Oryza sativa angiosperms tig2
YWNF scaffold 2049653 Hedwigia ciliata bryophyta tig2
LNSF scaffold 2066586 Hypnum subimponens bryophyta tig2
FFPD scaffold 2008261 Ceratodon purpureus bryophyta tig2
XP 024395675.1 Physcomitrium patens bryophyta tig2
HVBQ scaffold 2002478 Tetraphis pellucida bryophyta tig2
RCBT scaffold 2027702 Sphagnum palustre bryophyta tig2
YBQN scaffold 2127465 Odontoschisma prostratum marchantiophyta tig2
WZYK scaffold 2089586 Bazzania trilobata marchantiophyta tig2
HPXA scaffold 2020085 Ptilidium pulcherrimum marchantiophyta tig2
NRWZ scaffold 2097704 Metzgeria crassipilis marchantiophyta tig2
GJZN01034292.1 mougeotiopsis calospora zygnematophyceae tig2
WDCW scaffold 2046075 Mesotaenium endlicherianum zygnematophyceae tig2
GBG80941.1 Chara braunii charophyceae tig2
VQBJ scaffold 2011174 Coleochaete scutata coleochaetophyceae tig2
QPDY scaffold 2026583 Coleochaete irregularis coleochaetophyceae tig2
TGNL scaffold 2000439 Picocystis salinarum chlorophyta tig2
TNAW scaffold 2072542 Pyramimonas parkeae chlorophyta tig2
EGNB scaffold 2026708 Scourfieldia sp chlorophyta tig2
GHP04649.1 Pycnococcus provasolii chlorophyta tig2
CAI6002734.1 Closterium NIES65 zygnematophyceae tig2
AEKF scaffold 2048358 Penium margaritaceum zygnematophyceae tig2
ISHC scaffold 2007820 Staurastrum sebaldi zygnematophyceae tig2
OSX71690.1 Porphyra umbilicalis rhodophyta tig1
ZULJ scaffold 2071806 Porphyra yezoensis rhodophyta tig1
PXF49080.1 Gracilariopsis chorda rhodophyta tig1
OBUY scaffold 2000628 Porphyridium cruentum rhodophyta tig1
PVGP scaffold 2019906 Porphyridium purpureum rhodophyta tig1
RTLC scaffold 2027776 Rhodella violacea rhodophyta tig1
RSOF scaffold 2009376 Glaucosphaera vacuolata rhodophyta tig1
KAJ8904716.1 Rhodosorus marinus rhodophyta tig1
POOW scaffold 2012151 Glaucocystis nostochinearum glaucocystophyceae tig1
PQED scaffold 2054425 Gloeochaete wittrockiana glaucocystophyceae tig1
YTYU scaffold 2001279 Cyanophora paradoxa glaucocystophyceae tig1
WP 071454006.1 Gloeomargarita lithophora cyanobacteria TF
WP 230839797.1 Gloeobacter morelensis cyanobacteria TF
WP 011144179.1 Gloeobacter violaceus cyanobacteria TF
WP 041244210.1 Gloeobacter kilaueensis cyanobacteria TF
WP 218082614.1 Anthocerotibacter panamensis cyanobacteria TF
WP 287129553.1 Candidatus Cyanaurora vandensis cyanobacteria TF

Figure S1: Extended phylogenetic tree
Full phylogenetic tree with all sequences (organism name and NCBI-identifier) to infer the evolutionary history of chloroplast trigger factor.

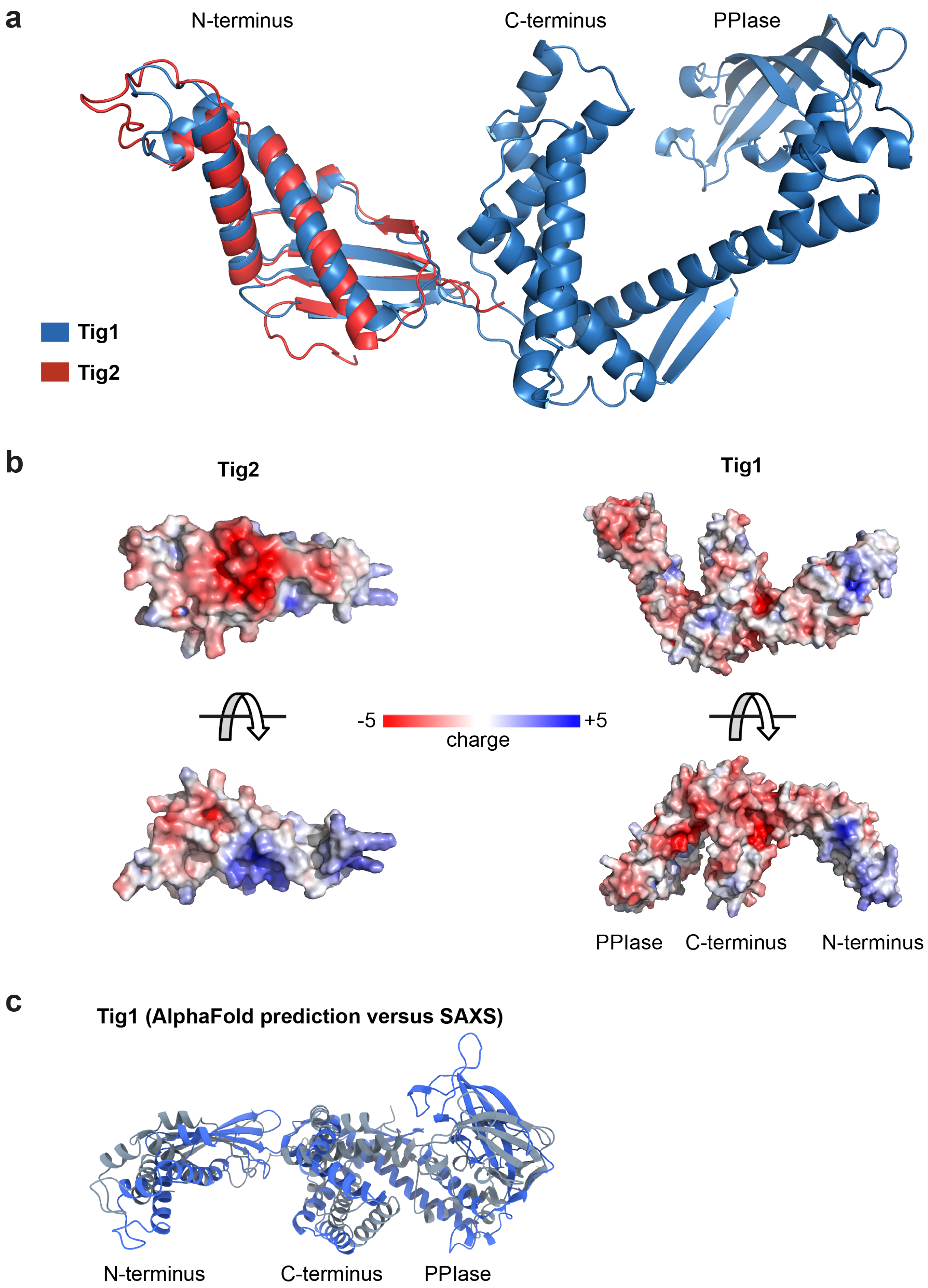

Figure S2: Conformational comparison between Tig1 and Tig2
a AlphaFold prediction of the conformation of the N-terminal domain of Tig1 (in blue) and the full-length sequences of Tig2 (in red). Conformations were superimposed by Chimera [1]. **b** Surface charge distribution of Tig1 and Tig2. **c** Comparison of the Tig1 AlphaFold prediction (blue) with the SREFLEX (version ATSAS 3.3.0; r14945, grey) model based on Arabidopsis Tig1 [2].

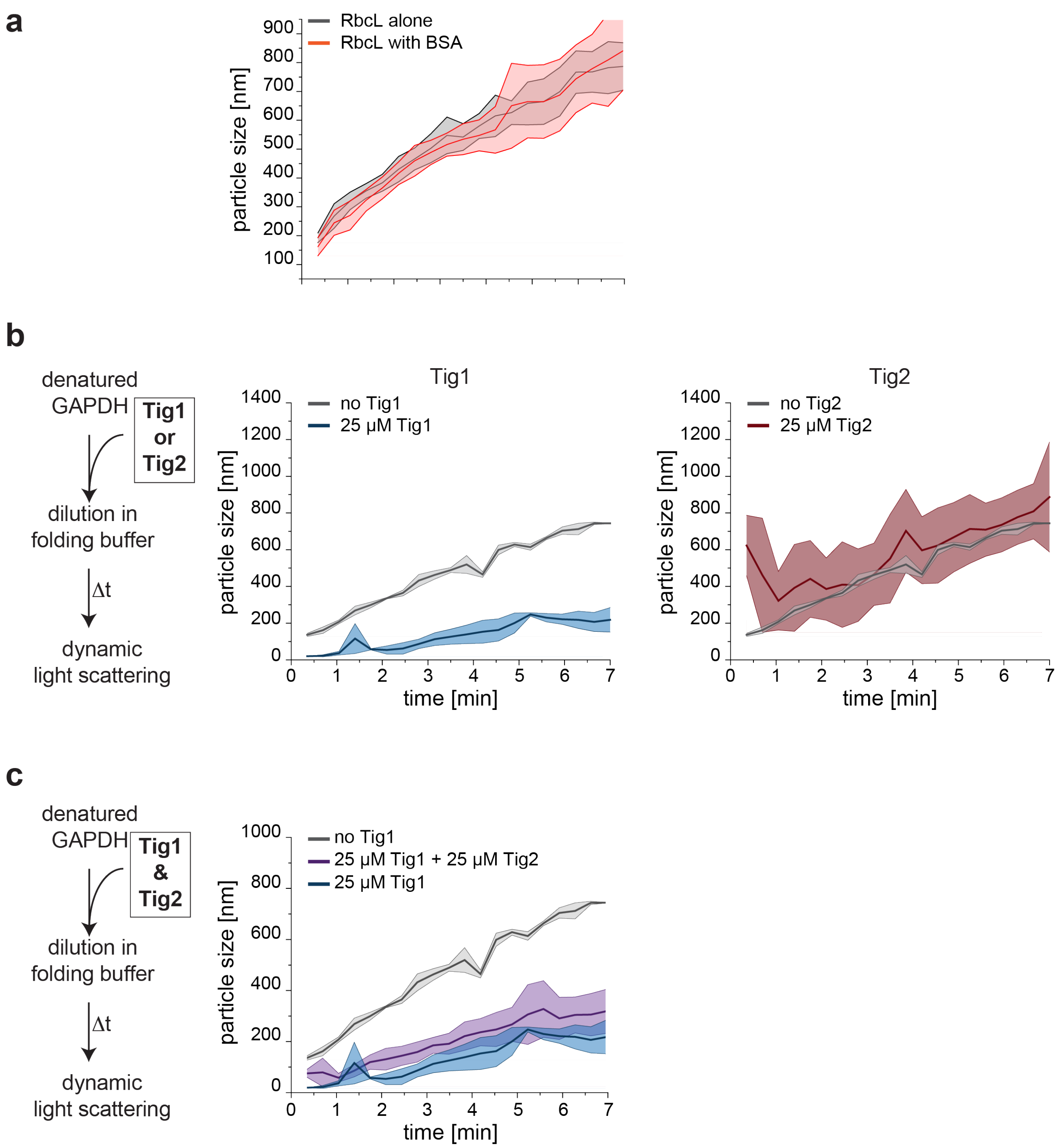

Figure S3: Prevention of GAPDH aggregation is achieved by Tig1 but not by Tig2
Chaperone activity assays and controls of Tig1 and Tig2. **a** Chemically denatured RbcL protein was diluted to 1 µM in folding puffer in the absence or presence of 25 µM purified BSA protein, respectively. Dynamic light scattering (DLS) was monitored over seven minutes at 25°C. **b** Left panel shows the experimental setup. Chemically denatured GAPDH protein was diluted to 2.5 µM in folding puffer in the absence or presence of 25 µM purified Tig1 or Tig2 protein, respectively. **c** Assay as in (b) in the presence of both chaperones. DLS was monitored over seven minutes at 25°C. Middle panel: Changes of hydrodynamic particle size (given as distribution widths of z-average diameters) in the absence or presence of Tig1. Right panel: DLS in the absence or presence of Tig2. Each data series represents the arithmetic mean values of 3-4 technical replicates and 1-2 biological replicates, deviations are displayed as ribbon plot.

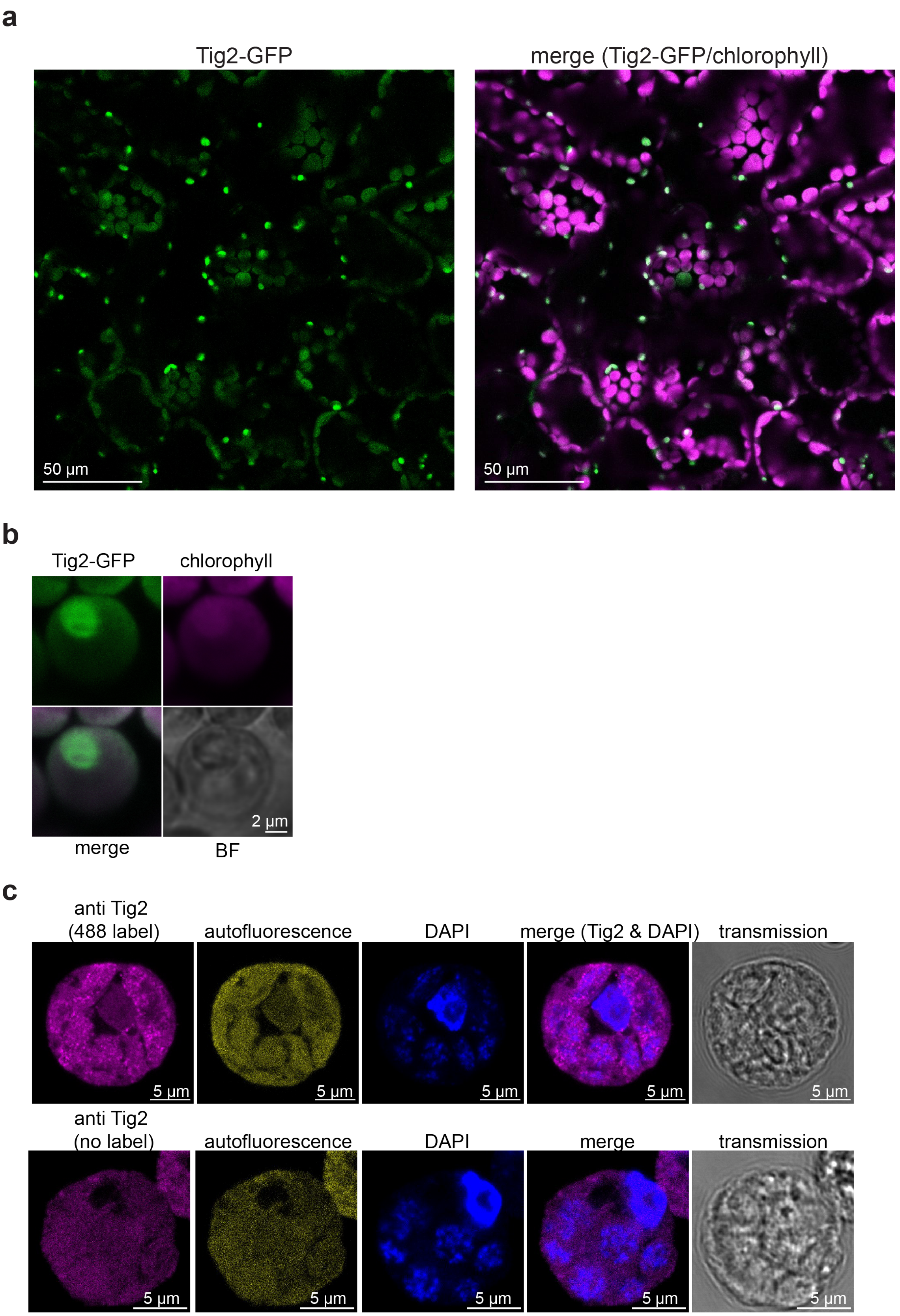

Figure S4: Chloroplast distribution of Tig2-GFP

a Overview confocal microscopy images of Tig2-GFP (green, left panel) and merge with chlorophyll autofluorescence (magenta, right panel) of Arabidopsis chloroplasts. Experiments were performed in two biological replicates with two independent lines. **b** Representative zoom-in image of a chloroplast with Tig2-GFP. **c** Immunofluorescence

microscopy of Arabidopsis protoplasts. Tig2 staining with antibody lacking the 488 label serves as negative control. Three independent biological replicates were done.

Tig1

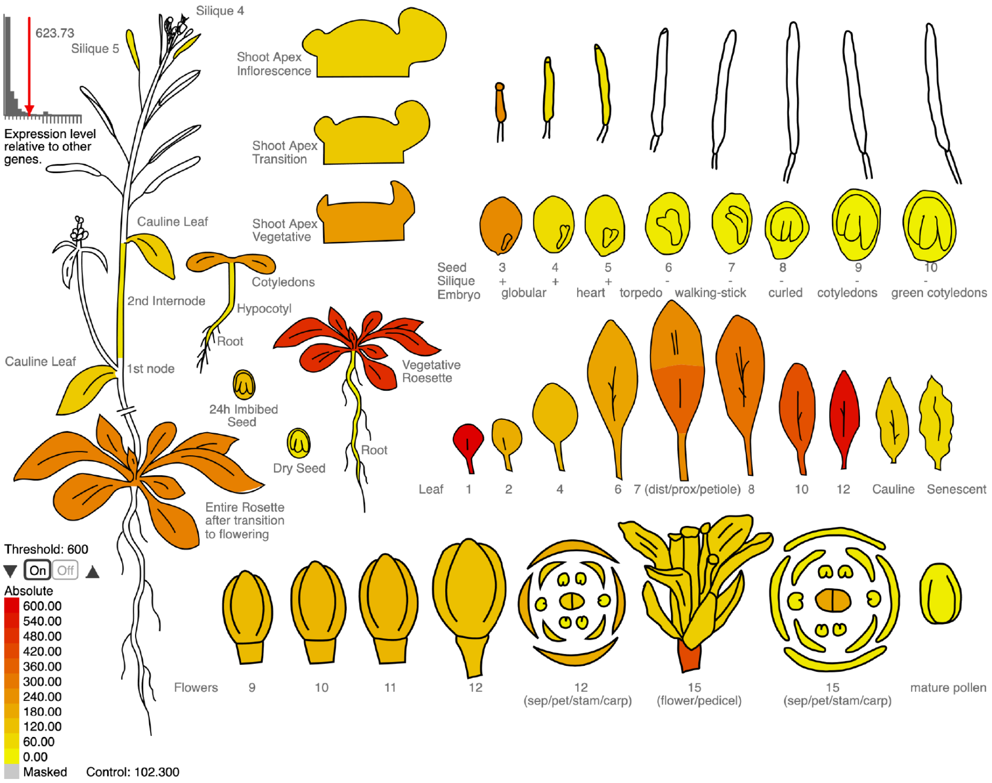

Tig2

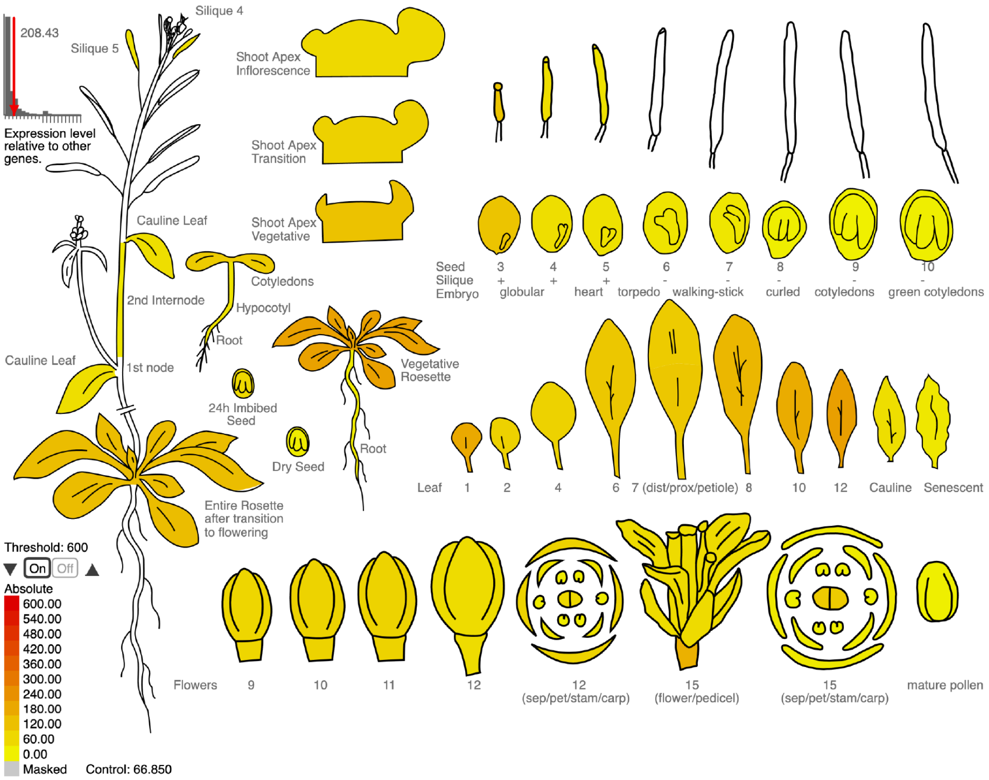

Figure S5: Tissue-specific expression of *Tig1* and *Tig2* in Arabidopsis.
Arabidopsis eFT Browser information[3] on tissue specific expression of *Tig1* (At5g55220, top panel) and *Tig2* (At2g30695, lower panel). This image was generated with the AtGenExpress eFP at https://bar.utoronto.ca/eplant by Waese et al[3]. Data were generated according to [4,5], with red = strongest relative expression and yellow = lowest relative expression.

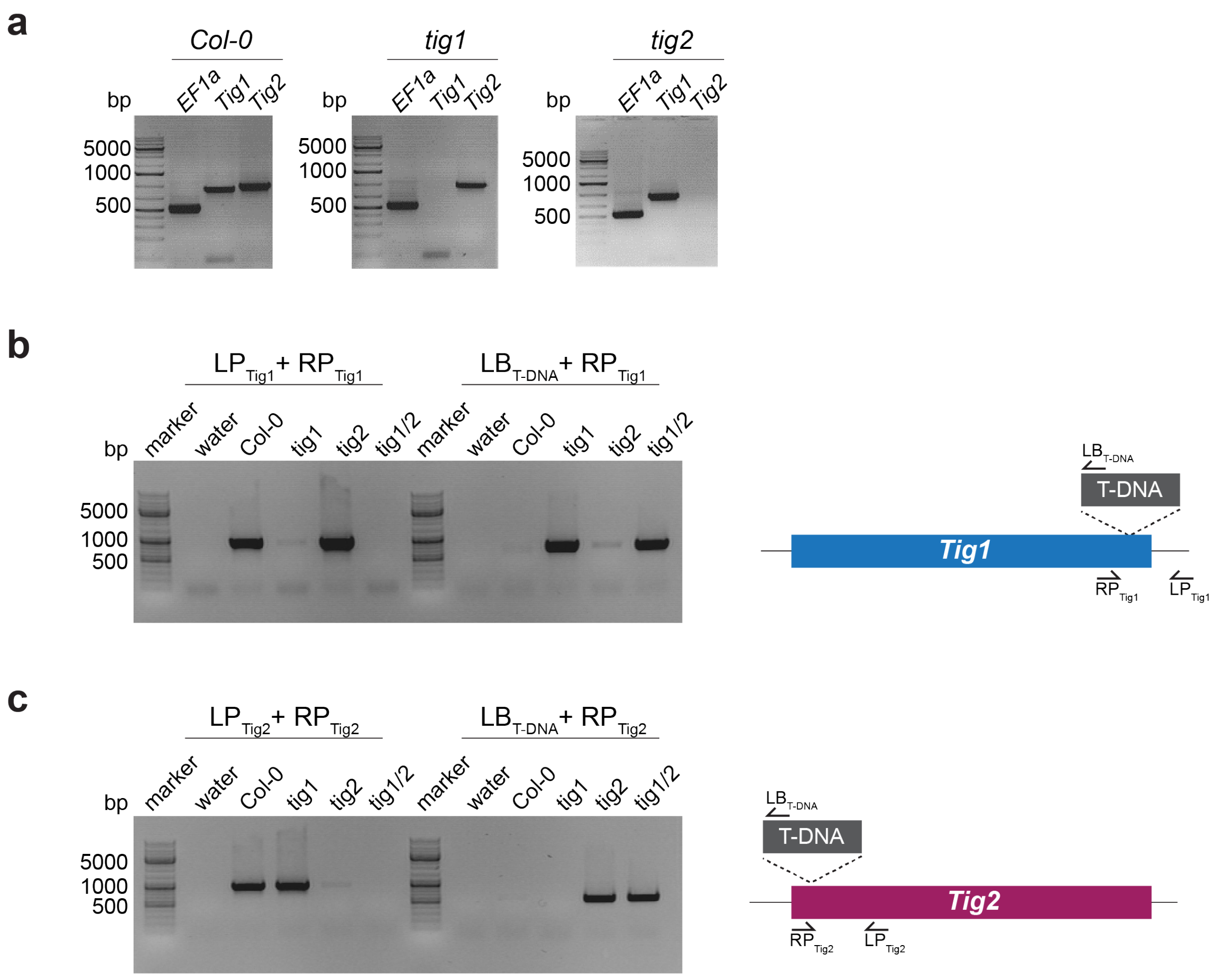

Figure S6: Validation of locus disruptions within trigger factor mutants
PCR over genomic DNA from Col-0, *tig1* and *tig2* mutants. **a** Agarose gels of 497 bp PCR product of *EF1*α (At5g60390), 900 bp PCR product over *TIG1* (At5g55220) and 963 bp PCR product over *TIG2* (At2g30695). **b** Agarose gels of PCR confirming disruption of the *TIG1* locus (left) and insertion of the T-DNA cassette (right). **c** Agarose gels of PCR confirming disruption of the *TIG2* locus (left) and insertion of the T-DNA cassette (right). Cartoon represents position of the insertion cassette and the used primers. All primer sequences are listed in Table S2. *tig1* mutant was characterized previously [6].

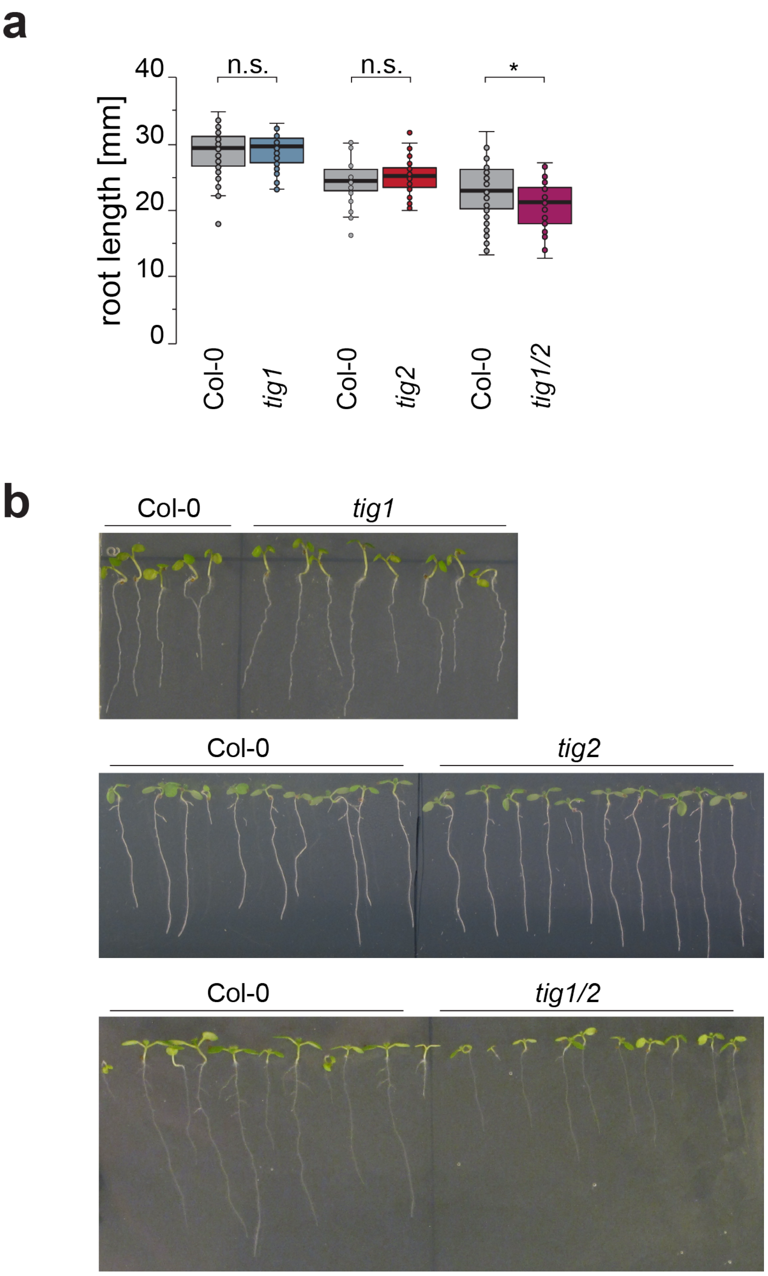

Figure S7: Trigger factor double mutants have a reduced root growth
a Root length of Arabidopsis wild-type (Col-0) and mutant lines, vertically grown on MS+ agar plates for 7 days after germination. Mutants are compared with the respective wild type, which were grown on the same plate. Boxplots show data of >37 individual plants grown in three independent biological replicates, respectively. Median is shown as solid line, box indicates lower and upper quartile, and the whiskers represent the data points that fall within 1.5 times the interquartile range (IQR) from the lower and upper quartiles. Any data point outside this range is considered as outlier. All samples are normally distributed after Kolmogorov-Smirnov, significant changes were determined by unpaired two-tailed Student's *t*-test. "n.s." = not significant, "*" = significance with $p = 0.02$. **b** Representative images of vertically grown seedlings (7 DAG).

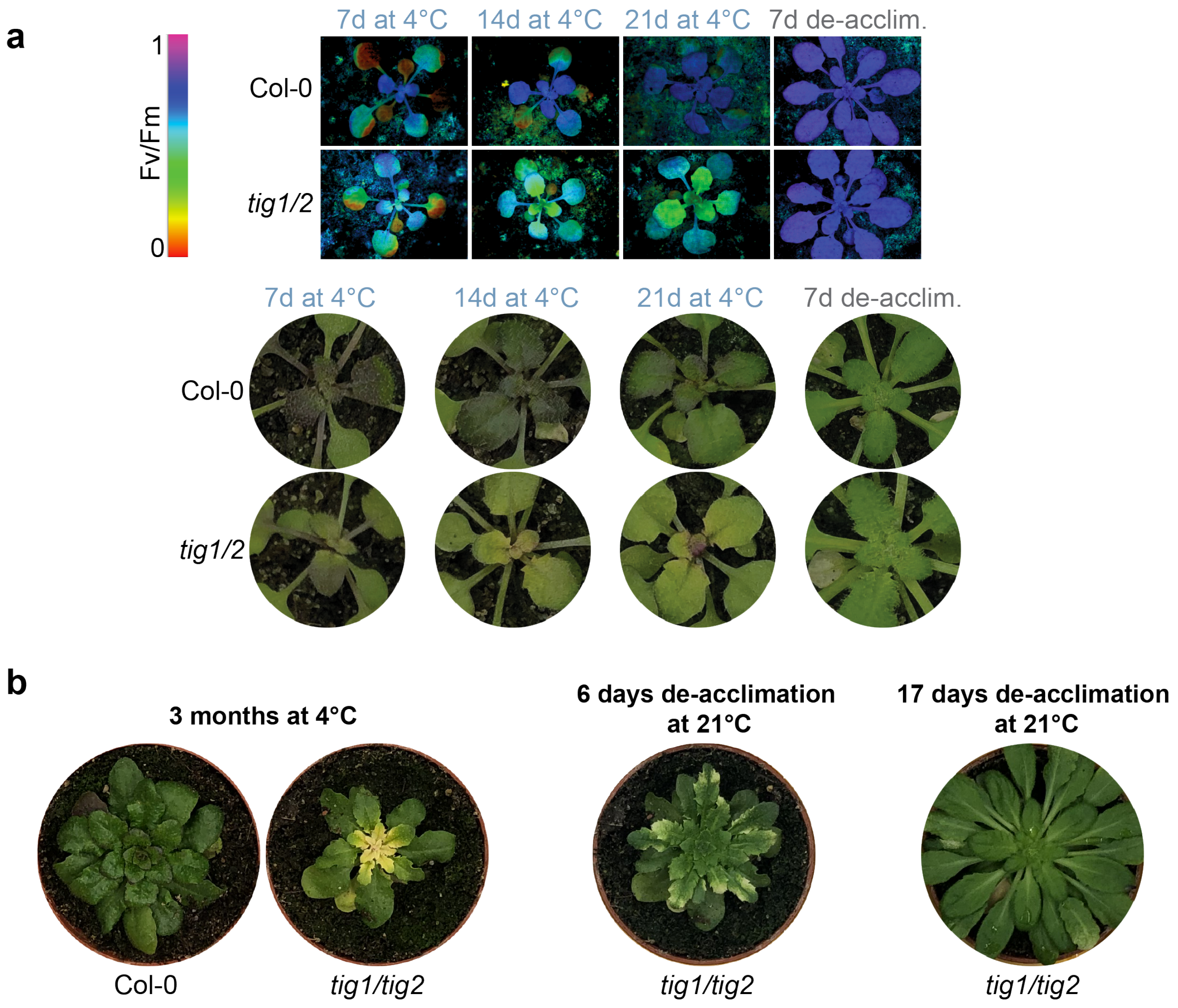

Figure S8: Phenotypes of cold-exposed trigger factor double mutants
a Top panel: Exemplary false-color images of Fv/Fm chlorophyll fluorescence of Col-0 and *tig1/2* lines. Lower panel: Images of respective rosettes. Corresponding maximum quantum yield of fluorescence (Fv/Fmax), representing activity of photosystem II are shown in Fig. 3e. **b** Reversible cold defect of Arabidopsis Col-0 and *tig1/2* plants, kept for three months at 4°C and subsequent de-acclimation for 6 days at 21°C. Images are representative for biological replicates.

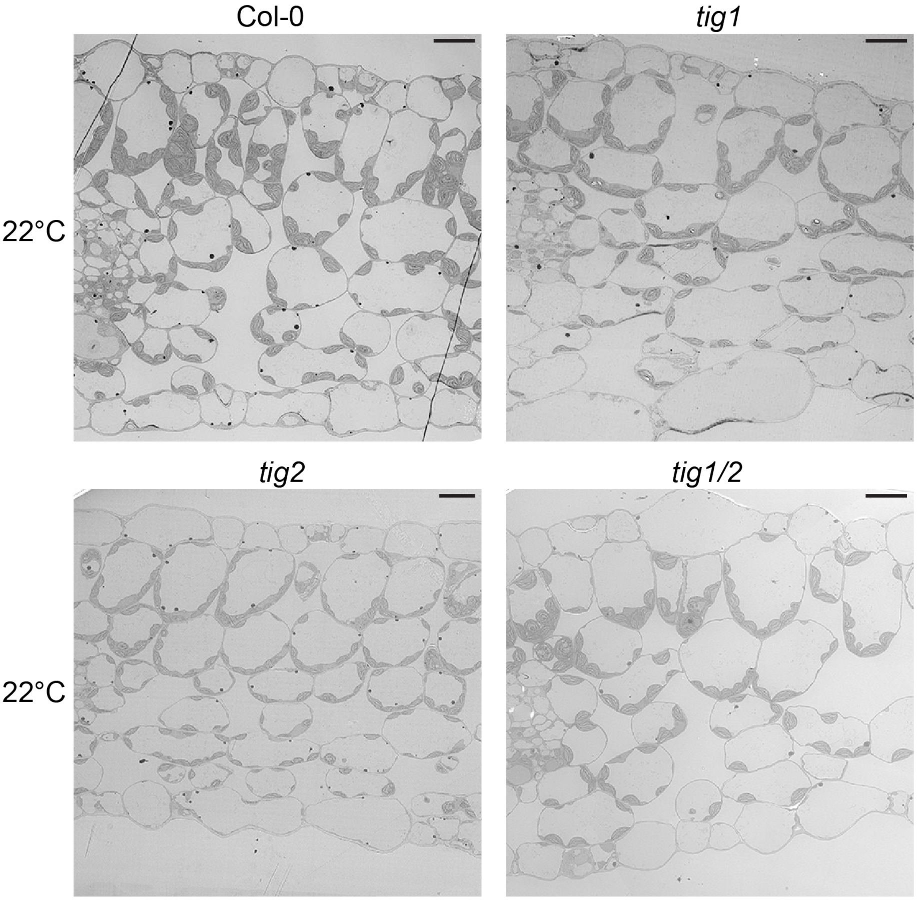
Col-0
tig1
22°C
tig2
tig1/2
22°C

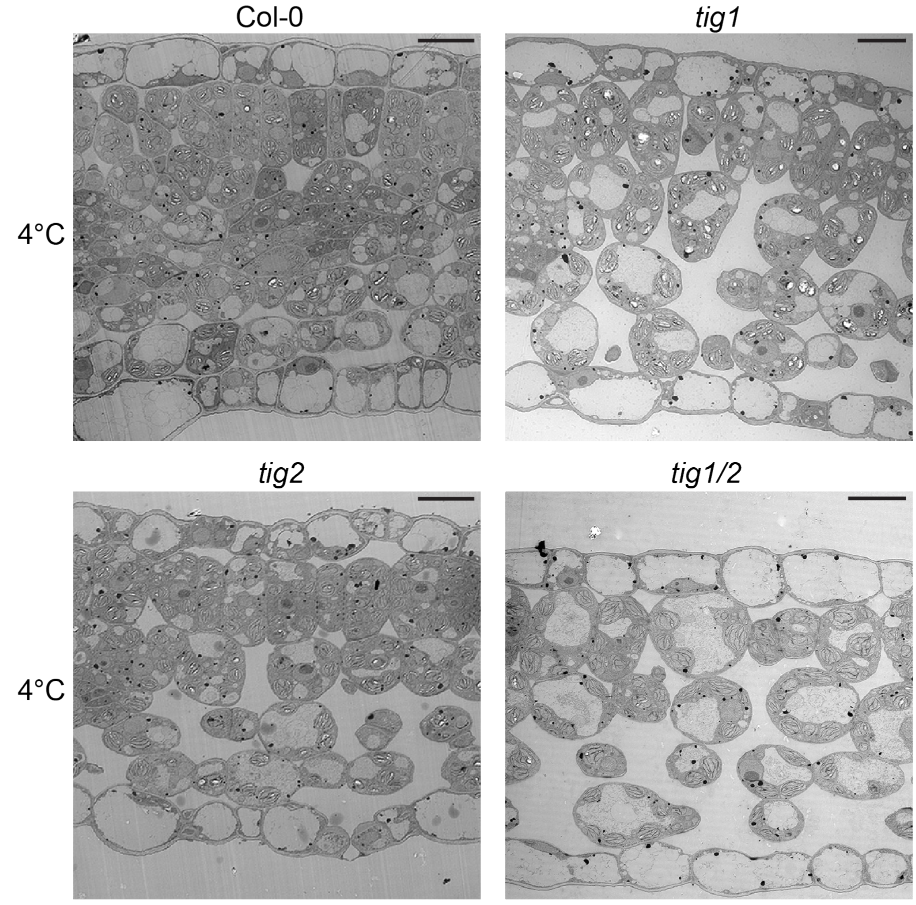
Col-0
tig1
4°C
tig2
tig1/2
4°C

Figure S9: Leaf cross-sections of Col-0 and trigger factor mutants
Transmission electron micrographs of ultra-thin sections (60 nm) from young leaves of 35 days old Arabidopsis plants. Plants were grown for 21 days at room temperature (22°C) and were kept for additional 14 days at these temperatures or were transferred for 14 days to 4°C. Leaf tissue from top to bottom: upper epidermis, palisade mesophyll, spongy mesophyll, lower epidermis. Scale bar represents 10 µm.

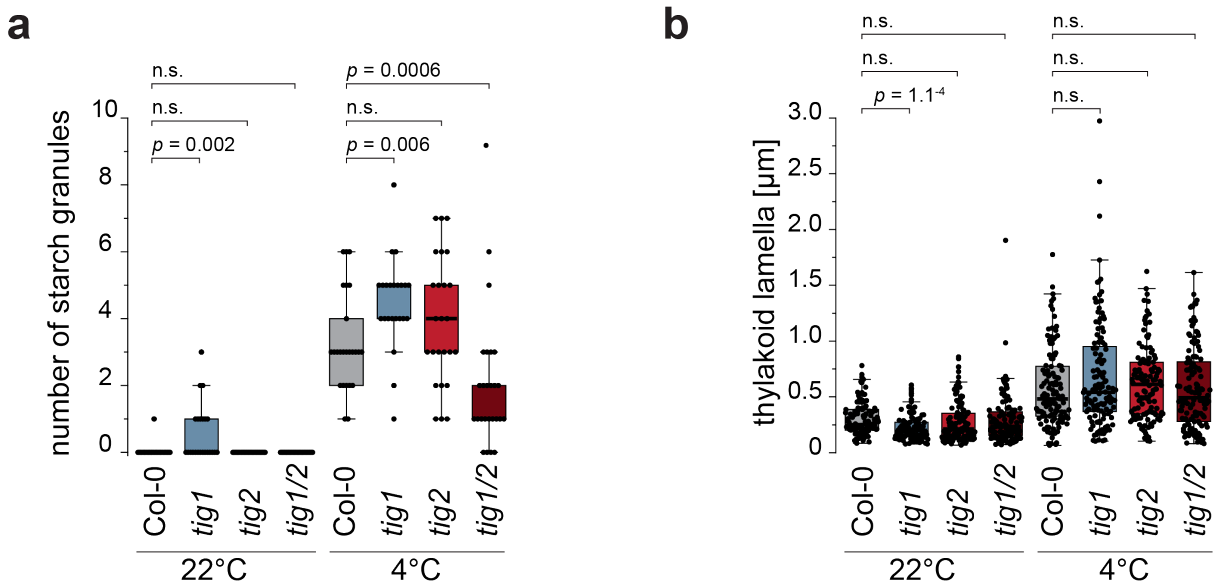

Figure S10: Starch granules and stroma lamellae length of Col-0 and trigger factor mutants

a Quantification of electron microscopy images (Fig. 4). Number of chloroplast-localized starch granules, derived from random quantification of 25 chloroplasts per Arabidopsis line and condition, respectively. **b** Length of stroma lamellae thylakoid membrane section determined for 5 sections in 25 chloroplasts per Arabidopsis line and condition, respectively. *P*-values of unpaired two-tailed Student's *t*-test are given. "n.s." = not significant. For all box plots, median is shown as solid line, box indicates lower and upper quartile, and the whiskers represent the data points that fall within 1.5 times the interquartile range (IQR) from the lower and upper quartiles. Any data point outside this range is considered as outlier.

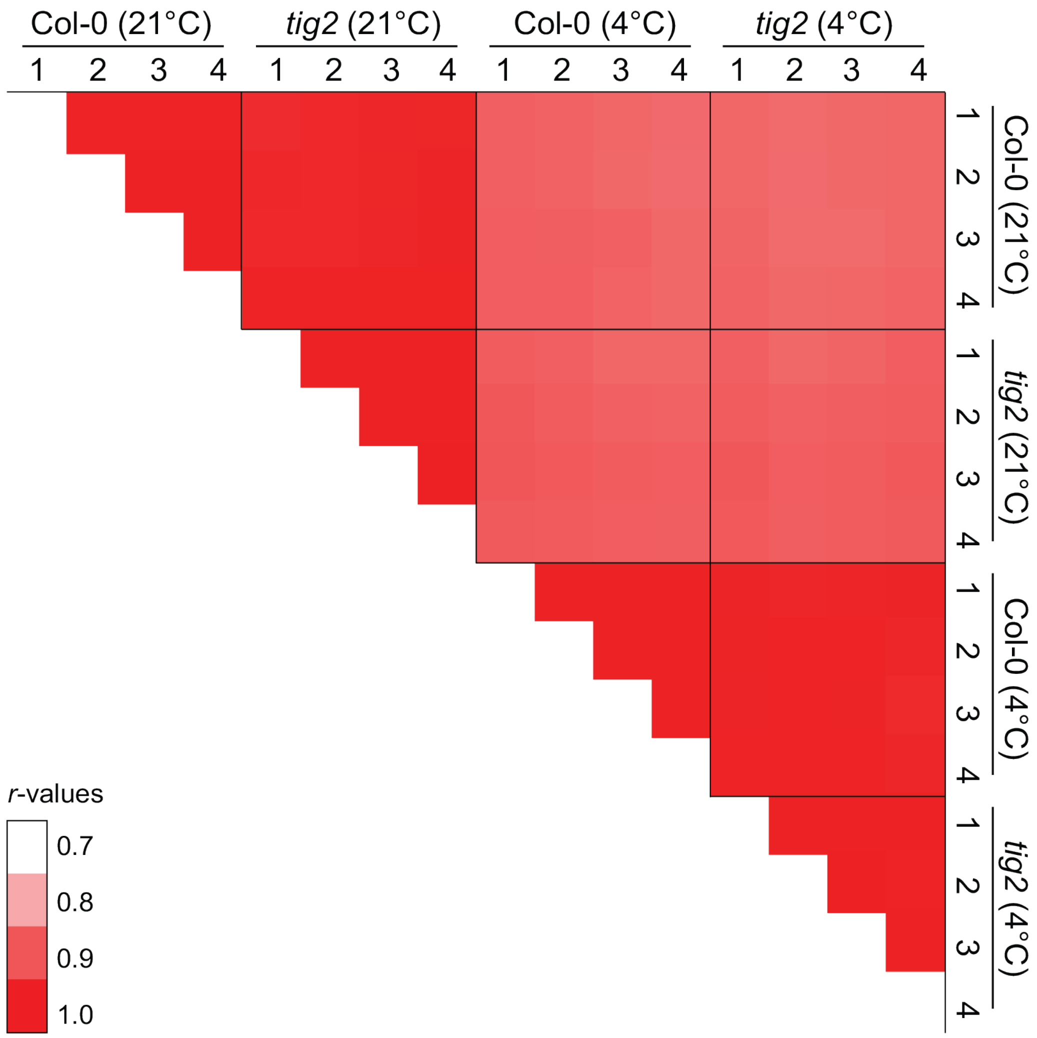

Figure S11: Reproducibility of proteomic data
Pearson correlation *r*-values between all whole-proteome mass spectrometry experiments from untreated and cold-treated Col-0 and *tig2* lines. Correlation is based on filtered log_2-transformed LFQ values set.

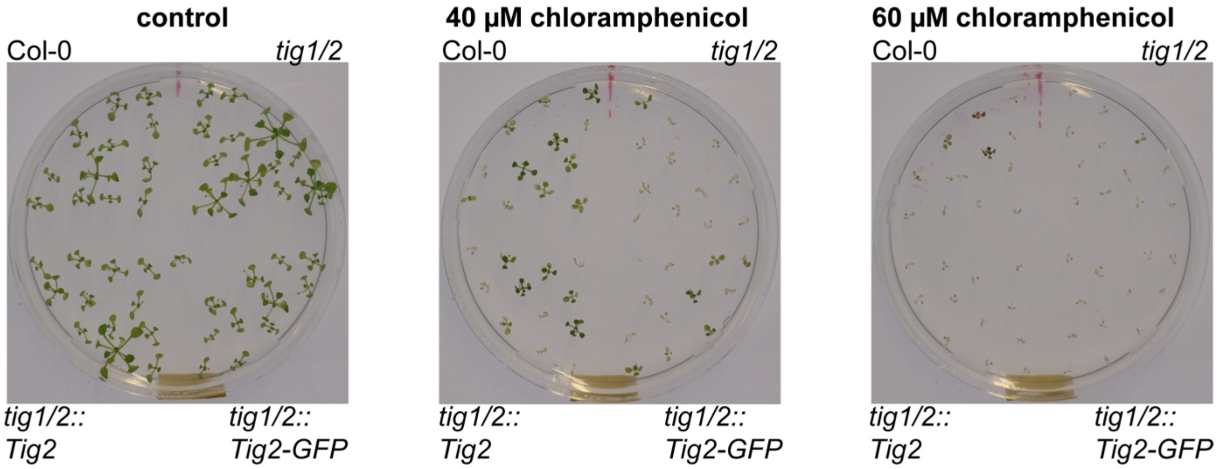

Figure S12: Reduced ribosome capacity in *tig2* mutants
Seedlings were grown for 10 days (DAG) on plates with 40 or 60 µM of chloramphenicol. Plates without chloramphenicol serve as control.

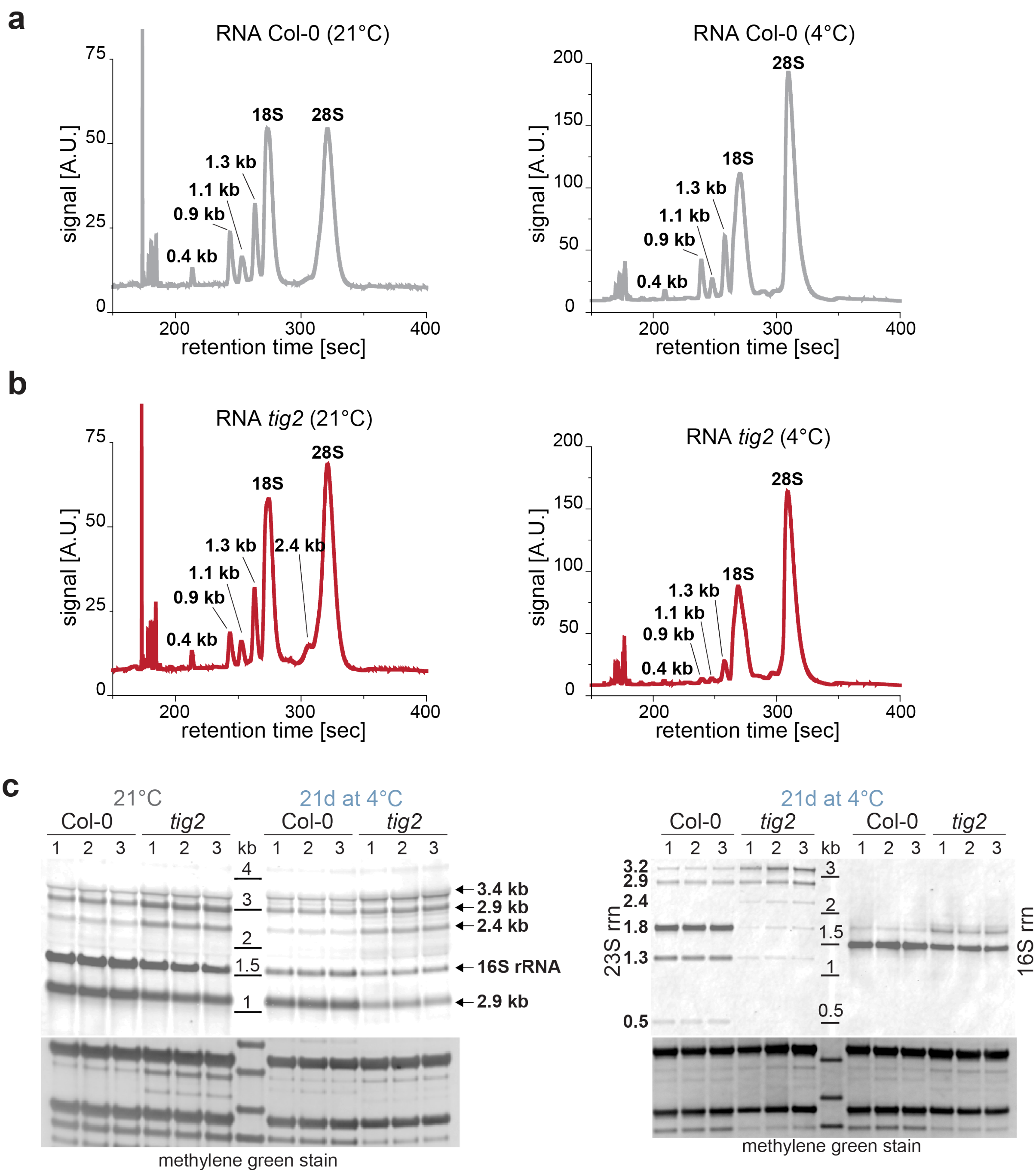

Figure S13: rRNA quality from Col-0 and *tig2* samples

Dominant rRNA fragments of isolated RNA from samples collected of Col-0 (**a**) and *tig2* mutant lines (**b**), exposed for the indicated times at 21°C and 4°C. **c** Top panel: Northern blot of RNA samples from (a) and (b), with probes targeting the indicated 23S and 16S chloroplast rRNA fragments. Samples from independent biological replicates are shown. Bottom panels: methylene green stain serving as loading control.

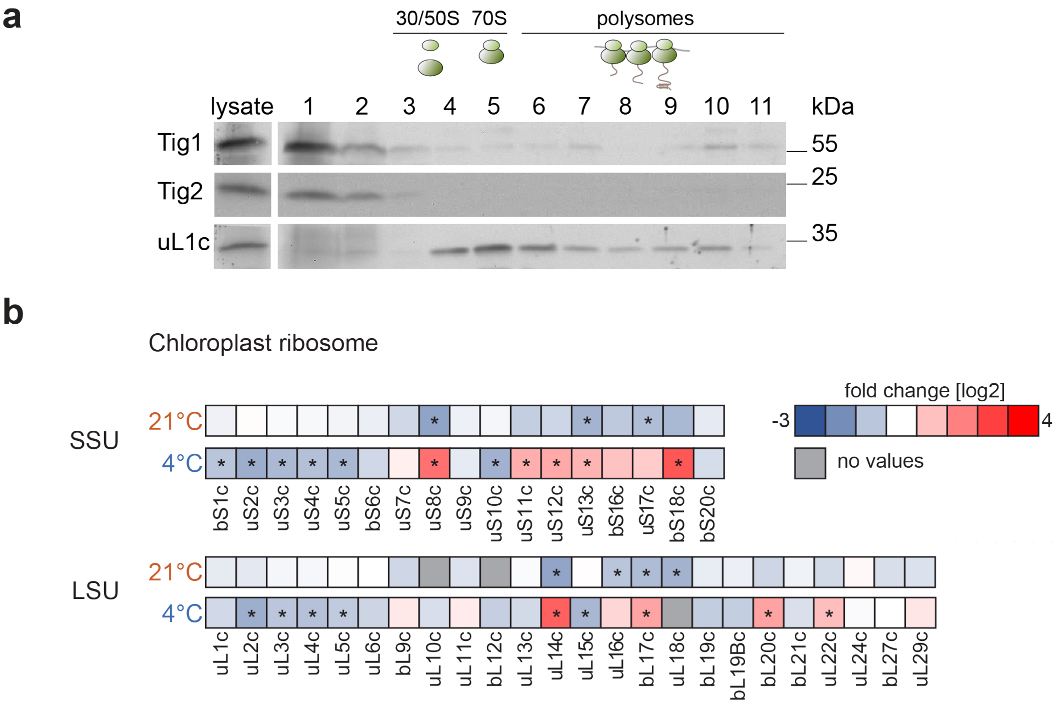

Figure S14: Polysome analysis and differential enrichment of cytosolic and plastidic ribosomal proteins between Col-0 and *tig2* samples

a Sucrose gradient fractionation and immunoblotting of polysomes in lysates from seedlings that were grown under standard growth conditions. Approximate positions of unassembled subunits including monosomes and polysomes in the gradient are illustrated by cartoons above the blots (n = 3). **b** Mass spectrometric quantification of ribosomal proteins from the small subunit (SSU) and the large subunit (LSU), respectively that were present in ribosomal pellets. Col-0 and *tig2 lines* were kept for 21 days in the cold (4°C) or at standard conditions (21°C) for one week. Heatmaps represent fold-change differences ($\log_2$) between Col-0 and *tig2* lines.

Supplementary Tables

Table S1. Trigger factor 1 and Trigger factor 2 sequences of selected plant species

Name[1]	Species	Gene Model	Accession Number (UniProt)[2]	N-domain[3]	Predicted Transit Peptide[4]
PpTIG1	Physcomitrium patens	Phpat.011G039900	A0A7I4A568	A_{100}-D_{234}	99
PpTIG2	Physcomitrium patens	Pp3c14_20300V3	A0A2K1JIJ9	A_{70}-A_{250}	69
SfTIG1	*Sphagnum fallax*	Sphfalx0093s0024		A_{93}-D_{227}	92
SfTIG2	*Sphagnum fallax*	Sphfalx0027s0120		I_{73}-S_{244}	72
AtTIG1	*Arabidopsis thaliana*	At5g55220	Q8S9L5	A_{77}-D_{214}	76
AtTIG2	*Arabidopsis thaliana*	At2g30695	Q945Q5	C_{55}-S_{198}	54
CgTIG1	*Capsella grandiflora*	Cagra.2897s0023		A_{80}-D_{217}	79
CgTIG2	*Capsella grandiflora*	Cagra.6622s0002		C_{55}-S_{199}	54
BdTIG1	*Brachypodium distachyon*	Bradi1g42820	I1GYS7	A_{59}-D_{192}	58
BdTIG2	*Brachypodium distachyon*	Bradi2g57530	A0A0Q3GIQ1	S_{63}-S_{249}	62
PtTIG1	*Populus trichocarpa*	Potri.015G065900	B9IEG6	S_{61}-D_{195}	60
PtTIG2	*Populus trichocarpa*	Potri.013G125500	A0A2K1Y4X4	A_{62}-T_{210}	61
ZmTIG1	*Zea mays*	GRMZM2G127393	K7TWH1	A_{62}-D_{195}	61
ZmTIG2	*Zea mays*	GRMZM2G109526	B4FYP9	S_{75}-E_{249}	74
OsTIG1	*Oryza sativa*	Os06g20320	A2YC62	A_{69}-D_{204}	68
OsTIG2	*Oryza sativa*	Os01g0894700	Q5JLV2	A_{62}-TS_{229}	58

[1] Names are according to [2]. TIG1 proteins represent proteins containing a N-terminal ribosome-binding-domain, a peptidyl–prolyl isomerase domain and a C-terminal chaperone domain.

[2] UniProt protein accession numbers are given only for sequences with a 100% match by BLAST search.

[3] Sequence of the predicted N-terminal domain is given as amino acid single letter code. The position was determined based on sequence alignments.

[4] Predicted transit peptide length was determined by TargetP and ChloroP and validated by alignments [7,8].

Table S2: Primers used for cloning in this study

Primer #	Used for cloning of	5´- 3´ Sequence
Tig2NdeI-F	Heterologous Tig2 expression	GGCCGCATATGTGTGCTGCACCATCAGATGT
Tig2EcoRI-R	Heterologous Tig2 expression	GGTGGGAATTCTCAACTCGCTTCTTGAAGCTTT
Salk_037730_LP	SALK037730 validation	GTCAGAGGGAAGATTAGTCC
Salk_037730_RP	SALK037730 validation	AGGTTGAATATGGTGCTGCAG
Salk_110999_LP	SALK110999 validation	TTGTACATGCACCTGCTCAAG
Salk_110999_RP	SALK110999 validation	TTCGTTTCATCTCCGACTCTC
LBb1.3(T-DNA)	SALK037730 and SALK110999 validation	ATTTTGCCGATTTCGGAAC
TIG1-GFP-F	Tig1-GFP expression	AACAGGTCTCAGGCTCAACAATGGAGCTCTGTGTTATCAGCACG
TIG1-GFP-R	Tig1-GFP expression	AACAGGTCTCTCTGAACGAGTGATGTATTGAATCTCGGCTCGG
TIG2-GFP-F	Tig2-GFP expression	AACAGGTCTCAGGCTCAACAATGCAGACAATCATCCACAGTCTCTC
TIG2-GFP-R	Tig2-GFP expression	AACAGGTCTCTCTGAACTCGCTTCTTGAAGCTTTATAGTAGC

Table S3: Antibodies used in this study

Target	Source	Reference
uL1c	own production	[2]
AtTig1	own production	[6]
AtTig2	own production, raised against the mature chloroplast Tig2 protein (lacking the 54 amino acids of the chloroplast transit peptide) from *Arabidopsis thaliana*	this study
GFP	mouse monoclonal antibody, Roche (#11 814 460 001)	-
PsaA	polyclonal rabbit antibody, Agrisera (#AS06 172)	-
uL12c	J.-D.Rochaix/S. Ramundo	[9]
PsbA/D1	polyclonal rabbit antibody, Agrisera (#AS05 084)	-
PbsC/CP43	polyclonal rabbit antibody, Agrisera (#AS11 1787)	-
RbcL	own production	[2]

References

1 Pettersen, E. F. *et al.* UCSF Chimera--a visualization system for exploratory research and analysis. *J Comput Chem* **25**, 1605-1612 (2004). https://doi.org:10.1002/jcc.20084

2 Ries, F. *et al.* Structural and molecular comparison of bacterial and eukaryotic trigger factors. *Sci Rep* **7**, 10680 (2017). https://doi.org:10.1038/s41598-017-10625-2

3 Waese, J. *et al.* ePlant: Visualizing and Exploring Multiple Levels of Data for Hypothesis Generation in Plant Biology. *Plant Cell* **29**, 1806-1821 (2017). https://doi.org:10.1105/tpc.17.00073

4 Nakabayashi, K., Okamoto, M., Koshiba, T., Kamiya, Y. & Nambara, E. Genome-wide profiling of stored mRNA in Arabidopsis thaliana seed germination: epigenetic and genetic regulation of transcription in seed. *Plant J* **41**, 697-709 (2005). https://doi.org:10.1111/j.1365-313X.2005.02337.x

5 Schmid, M. *et al.* A gene expression map of Arabidopsis thaliana development. *Nat Genet* **37**, 501-506 (2005). https://doi.org:10.1038/ng1543

6 Rohr, M. *et al.* The role of plastidic trigger factor serving protein biogenesis in green algae and land plants. *Plant Physiol* (2019). https://doi.org:10.1104/pp.18.01252

7 Emanuelsson, O., Nielsen, H., Brunak, S. & von Heijne, G. Predicting subcellular localization of proteins based on their N-terminal amino acid sequence. *J Mol Biol* **300**, 1005-1016 (2000). https://doi.org:10.1006/jmbi.2000.3903

8 Emanuelsson, O., Nielsen, H. & von Heijne, G. ChloroP, a neural network-based method for predicting chloroplast transit peptides and their cleavage sites. *Protein Sci* **8**, 978-984 (1999). https://doi.org:10.1110/ps.8.5.978

9 Ramundo, S. *et al.* Conditional Depletion of the Chlamydomonas Chloroplast ClpP Protease Activates Nuclear Genes Involved in Autophagy and Plastid Protein Quality Control. *Plant Cell* **26**, 2201-2222 (2014). https://doi.org:10.1105/tpc.114.124842
