# Supplementary material for: A truncated variant of the ribosome-associated trigger factor specifically contributes to plant chloroplast ribosome biogenesis
Source: Nat Commun. 2025 Jan 13;16:629. doi: 10.1038/s41467-025-55813-1 (PMC11731035; doi:10.1038/s41467-025-55813-1)
Supplement: Supplementary file 3 — Description of Additional Supplementary Files [file 41467_2025_55813_MOESM3_ESM.pdf]

<u>Description of Additional Supplementary Files</u>

Supplementary Data 1: Proteomics data with identified and quantified proteins.

Tab 1: Comparison of whole cell lysate from Col-0 and tig2 lines grown at 22°C or exposed for 3 weeks at 4°C.

Tab 2: Comparison of proteins that were identified in ribosomal pellets of Col-0 and tig2 lines after exposure for 3 weeks at 4°C. Ribosomal pellets resulted from ultracentrifugation of cell lysate through a 25% sucrose cushion.

Tab 3: Comparison of proteins that were identified in ribosomal pellets of Col-0 and tig2, grown at 22°C. Ribosomal pellets resulted from ultracentrifugation of cell lysate through a 25% sucrose cushion.
