# Supplementary material for: A truncated variant of the ribosome-associated trigger factor specifically contributes to plant chloroplast ribosome biogenesis
Source: Nat Commun. 2025 Jan 13;16:629. doi: 10.1038/s41467-025-55813-1 (PMC11731035; doi:10.1038/s41467-025-55813-1)
Supplement: Supplementary file 5 — Reporting Summary [file 41467_2025_55813_MOESM5_ESM.pdf]

nature portfolio

Corresponding author(s): Felix Willmund

Last updated by author(s): Dec 2, 2024

Reporting Summary

Nature Portfolio wishes to improve the reproducibility of the work that we publish. This form provides structure for consistency and transparency in reporting. For further information on Nature Portfolio policies, see our Editorial Policies and the Editorial Policy Checklist.

Statistics

For all statistical analyses, confirm that the following items are present in the figure legend, table legend, main text, or Methods section.

n/a	Confirmed	
☐	☒	The exact sample size (n) for each experimental group/condition, given as a discrete number and unit of measurement
☐	☒	A statement on whether measurements were taken from distinct samples or whether the same sample was measured repeatedly
☐	☒	The statistical test(s) used AND whether they are one- or two-sided<br>*Only common tests should be described solely by name; describe more complex techniques in the Methods section.*
☒	☐	A description of all covariates tested
☐	☒	A description of any assumptions or corrections, such as tests of normality and adjustment for multiple comparisons
☐	☒	A full description of the statistical parameters including central tendency (e.g. means) or other basic estimates (e.g. regression coefficient) AND variation (e.g. standard deviation) or associated estimates of uncertainty (e.g. confidence intervals)
☐	☒	For null hypothesis testing, the test statistic (e.g. F, t, r) with confidence intervals, effect sizes, degrees of freedom and P value noted<br>*Give P values as exact values whenever suitable.*
☒	☐	For Bayesian analysis, information on the choice of priors and Markov chain Monte Carlo settings
☒	☐	For hierarchical and complex designs, identification of the appropriate level for tests and full reporting of outcomes
☐	☒	Estimates of effect sizes (e.g. Cohen's d, Pearson's r), indicating how they were calculated

Our web collection on statistics for biologists contains articles on many of the points above.

Software and code

Policy information about availability of computer code

Data collection	Collection was conducted with Microsoft Excel and Origin, Figures were assembled with Illustrator
Data analysis	Microscopy images were analyzed by the ZEN blue 2.3 or Fiji software and Lightbox 2024; Proteomics were analysed with Perseus

For manuscripts utilizing custom algorithms or software that are central to the research but not yet described in published literature, software must be made available to editors and reviewers. We strongly encourage code deposition in a community repository (e.g. GitHub). See the Nature Portfolio guidelines for submitting code & software for further information.

Data

Policy information about availability of data

All manuscripts must include a data availability statement. This statement should provide the following information, where applicable:

- Accession codes, unique identifiers, or web links for publicly available datasets
- A description of any restrictions on data availability
- For clinical datasets or third party data, please ensure that the statement adheres to our policy

Source data are provided with this paper. Proteomics data are available via ProteomeExchange consortium (http://www.ebi.ac.uk/pride) with the following identifier:
PXD057252 Data Set 1 - Global proteome of Col-0 and tig2 mutant

PXD057264 Data Set 2 – Analysis of sedimented ribosomes
All gene accession numbers are given in the Supplementary Data and are according to the Arabidopsis standard https://www.arabidopsis.org.

Research involving human participants, their data, or biological material

Policy information about studies with human participants or human data. See also policy information about sex, gender (identity/presentation), and sexual orientation and race, ethnicity and racism.

Reporting on sex and gender	N/A
Reporting on race, ethnicity, or other socially relevant groupings	N/A
Population characteristics	N/A
Recruitment	N/A
Ethics oversight	N/A

Note that full information on the approval of the study protocol must also be provided in the manuscript.

Field-specific reporting

Please select the one below that is the best fit for your research. If you are not sure, read the appropriate sections before making your selection.

☒ Life sciences ☐ Behavioural & social sciences ☐ Ecological, evolutionary & environmental sciences

For a reference copy of the document with all sections, see nature.com/documents/nr-reporting-summary-flat.pdf

Life sciences study design

All studies must disclose on these points even when the disclosure is negative.

Sample size	In general 3-4 biological replicates (individual plants from different pots) were collected. In addition, technical replicates (e.g. > 30 seedlings for size determination or multiple leaves with randomized sections for photosynthesis measurements) were measured.
Data exclusions	For proteomic analyses, peptide counts were excluded if they were found in less than 3 o the 4 biological replicates. For all other experiments no data were excluded
Replication	For proteomic analyses, 4 biological replicates (plants from different pots) were used. For Immunoblot three independent experiments were performed. Microscopy was performed for multiple leaves from several plants (>3) per condition and line.
Randomization	Plant growth and harvesting was randomized to exclude positional effects during growth.
Blinding	N/A

Reporting for specific materials, systems and methods

We require information from authors about some types of materials, experimental systems and methods used in many studies. Here, indicate whether each material, system or method listed is relevant to your study. If you are not sure if a list item applies to your research, read the appropriate section before selecting a response.

Materials & experimental systems

n/a	Involved in the study	
☐	☒	Antibodies
☒	☐	Eukaryotic cell lines
☒	☐	Palaeontology and archaeology
☒	☐	Animals and other organisms
☒	☐	Clinical data
☒	☐	Dual use research of concern
☐	☒	Plants

Methods

n/a	Involved in the study	
☒	☐	ChIP-seq
☒	☐	Flow cytometry
☒	☐	MRI-based neuroimaging

Antibodies

Antibodies used	All antibodies are listed in Supplemental Table S3. uL1c, Tig1, Tig2, RbcL are from own production. PsaA, PsbA, PsbC are from Agrisera. uL12c is a gift from RD Rochaix, GFP is purchased from Roche.
Validation	Commercially antibodies passed validation. Own antibody were tested against their epitope and in respective mutants (for lacking signal) if applicable. uL1, Tig1 and RbcL antibodies were published earlier (references are given in the manuscript)

Plants

Seed stocks	Seeds were obtained from the Nottingham Arabidopsis Stock Centre
Novel plant genotypes	Double mutant was generated by crossing.<br>Complementing lines and lines with tagged GFP were transformed into Agrobacterium tumefaciens (GV3101 containing pMP90)
Authentication	All lines were examined by PCR over the respective locus and sequencing. In addition immoblots and proteomics confirmed deletions
