# Supplementary material for: A truncated variant of the ribosome-associated trigger factor specifically contributes to plant chloroplast ribosome biogenesis
Source: Nat Commun. 2025 Jan 13;16:629. doi: 10.1038/s41467-025-55813-1 (PMC11731035; doi:10.1038/s41467-025-55813-1)
Supplement: Supplementary file 6 — Source Data [file 41467_2025_55813_MOESM6_ESM.zip › Source Data/uncropped immunoblots.pptx]

## Slide 1
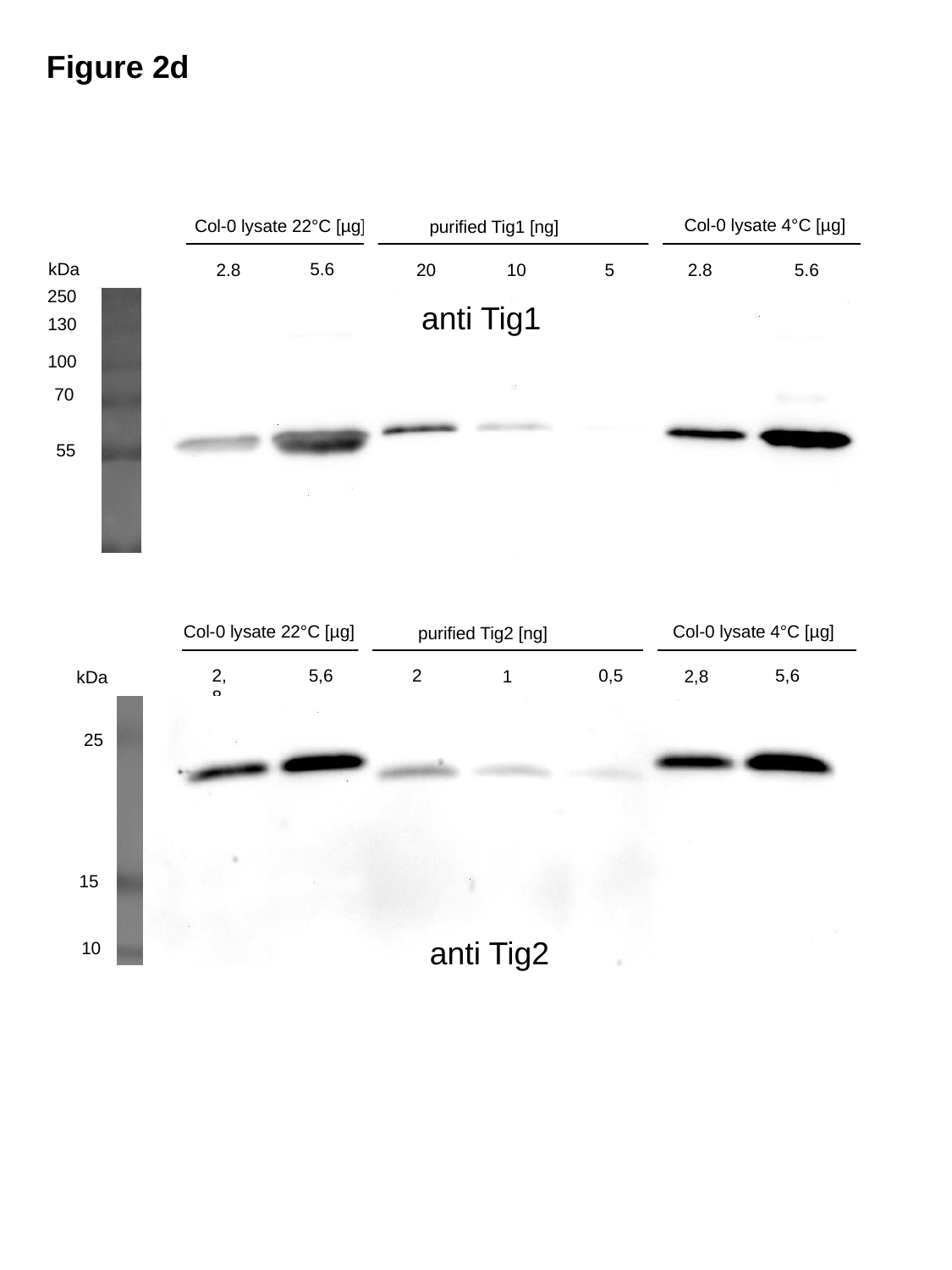

Figure 2d
Col-0 lysate 4°C [µg]
Col-0 lysate 22°C [µg]
purified Tig1 [ng]
5.6
kDa
20
2.8
5
5.6
2.8
10
250
anti Tig1
130
100
70
55
Col-0 lysate 4°C [µg]
Col-0 lysate 22°C [µg]
purified Tig2 [ng]
5,6
5,6
2
2,8
0,5
2,8
1
kDa
25
15
anti Tig2
10

## Slide 2
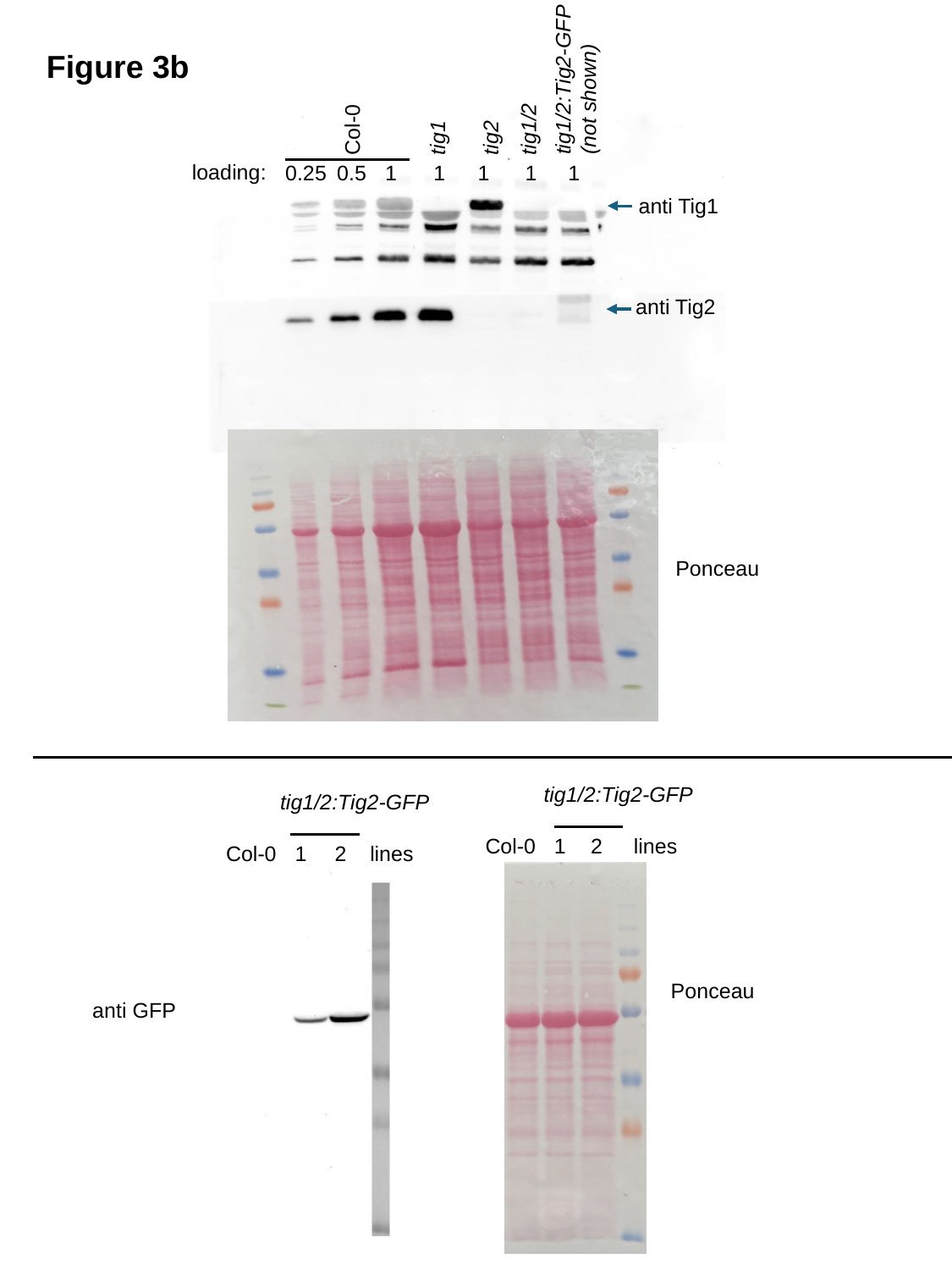

Figure 3b
tig1/2:Tig2-GFP
(not shown)
tig1/2
Col-0
tig1
tig2
loading:
0.25
0.5
1
1
1
1
1
anti Tig1
anti Tig2
Ponceau
tig1/2:Tig2-GFP
tig1/2:Tig2-GFP
lines
1
2
Col-0
lines
1
2
Col-0
Ponceau
anti GFP

## Slide 3
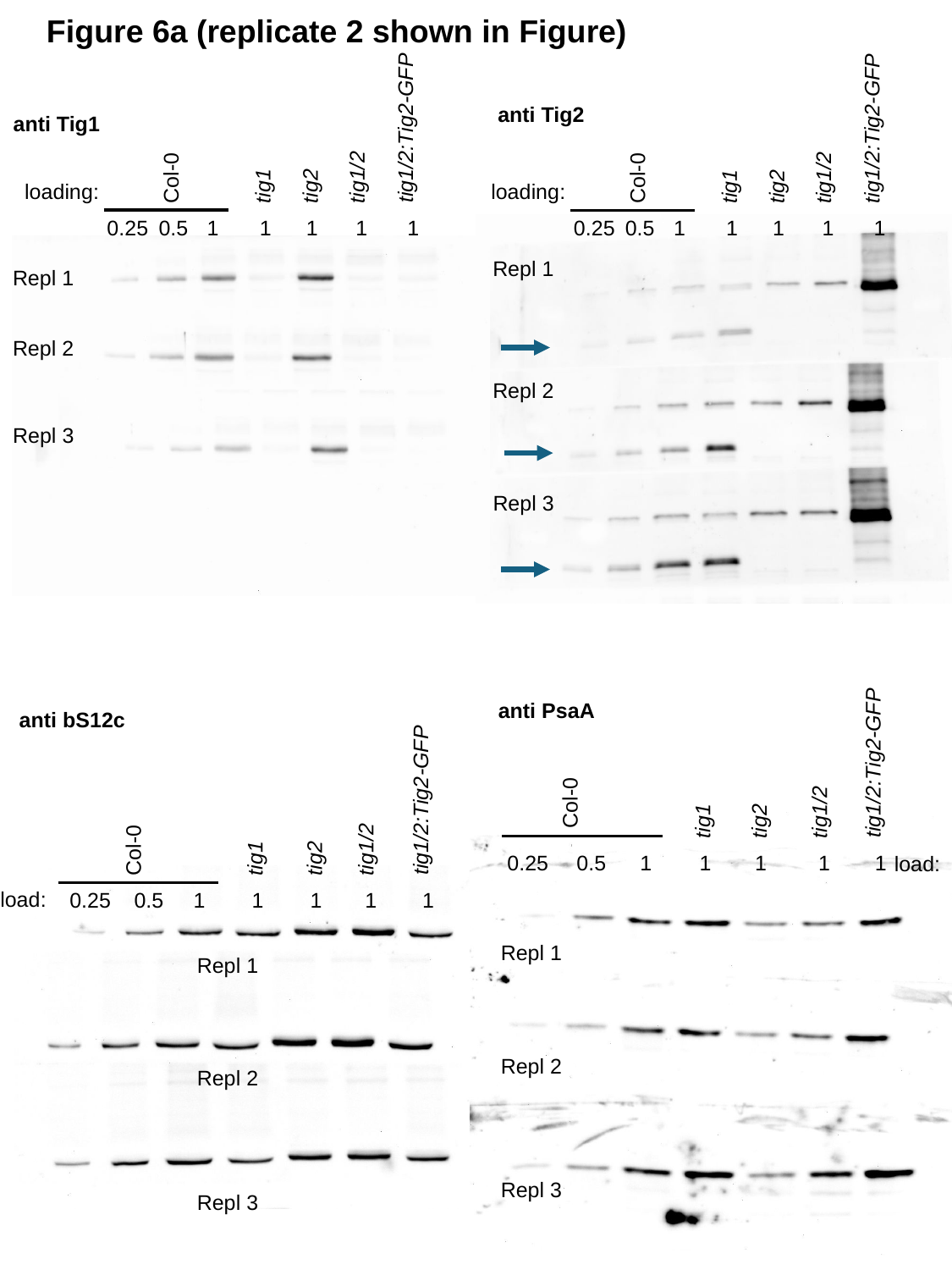

Figure 6a (replicate 2 shown in Figure)
anti Tig2
anti Tig1
tig1/2:Tig2-GFP
tig1/2:Tig2-GFP
tig1/2
tig1/2
Col-0
Col-0
tig1
tig2
tig1
tig2
loading:
loading:
0.25
0.5
1
1
1
1
1
0.25
0.5
1
1
1
1
1
Repl 1
Repl 1
Repl 2
Repl 2
Repl 3
Repl 3
anti PsaA
anti bS12c
tig1/2:Tig2-GFP
tig1/2:Tig2-GFP
Col-0
tig1/2
tig1
tig2
tig1/2
Col-0
tig1
tig2
0.25
0.5
1
1
1
1
1
load:
load:
0.25
0.5
1
1
1
1
1
Repl 1
Repl 1
Repl 2
Repl 2
Repl 3
Repl 3

## Slide 4
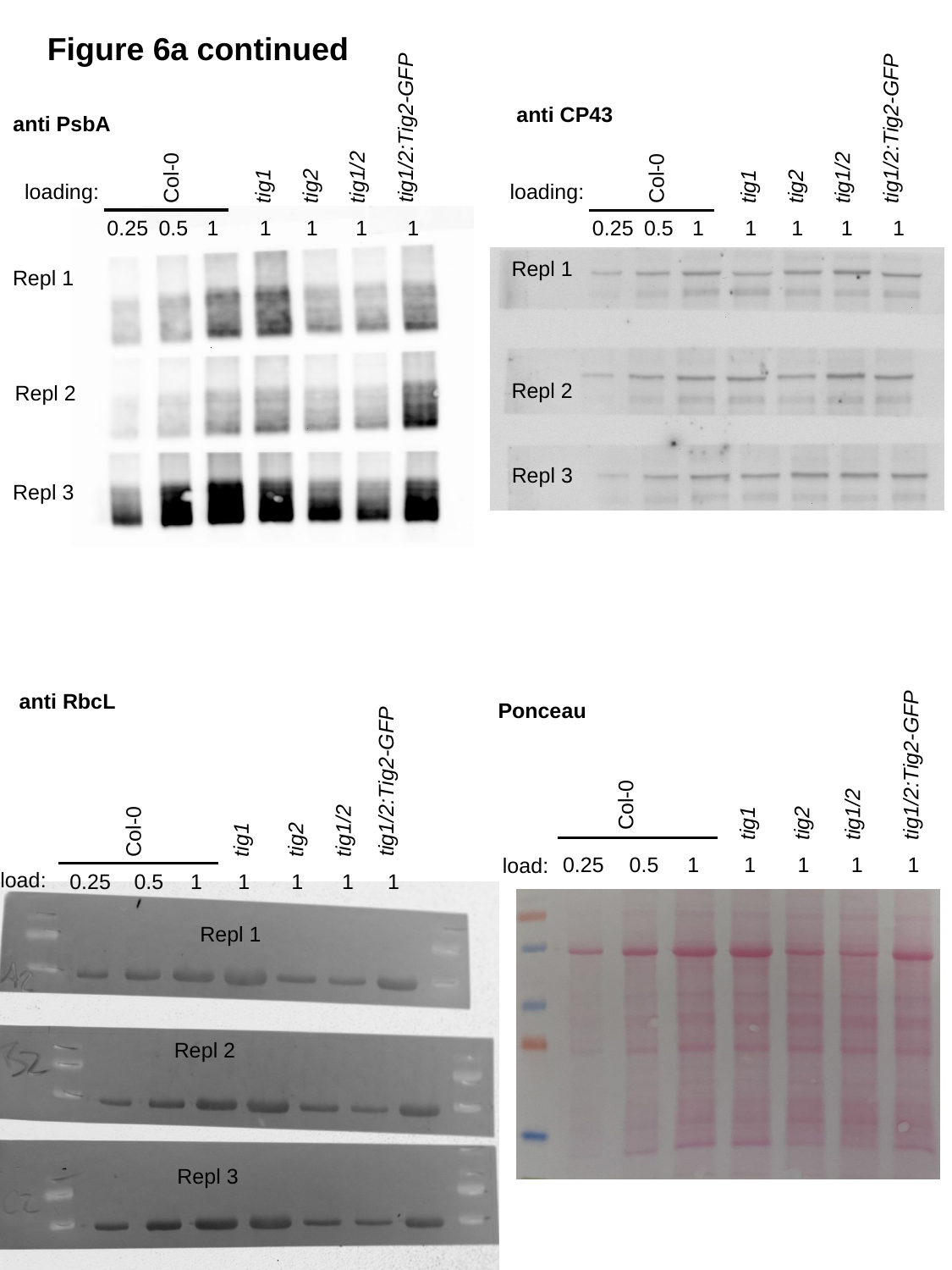

Figure 6a continued
anti CP43
anti PsbA
tig1/2:Tig2-GFP
tig1/2:Tig2-GFP
tig1/2
tig1/2
Col-0
Col-0
tig1
tig2
tig1
tig2
loading:
loading:
0.25
0.5
1
1
1
1
1
0.25
0.5
1
1
1
1
1
Repl 1
Repl 1
Repl 2
Repl 2
Repl 3
Repl 3
anti RbcL
Ponceau
tig1/2:Tig2-GFP
tig1/2:Tig2-GFP
Col-0
tig1/2
tig1
tig2
tig1/2
Col-0
tig1
tig2
0.25
0.5
1
1
1
1
1
load:
load:
0.25
0.5
1
1
1
1
1
Repl 1
Repl 2
Repl 3

## Slide 5
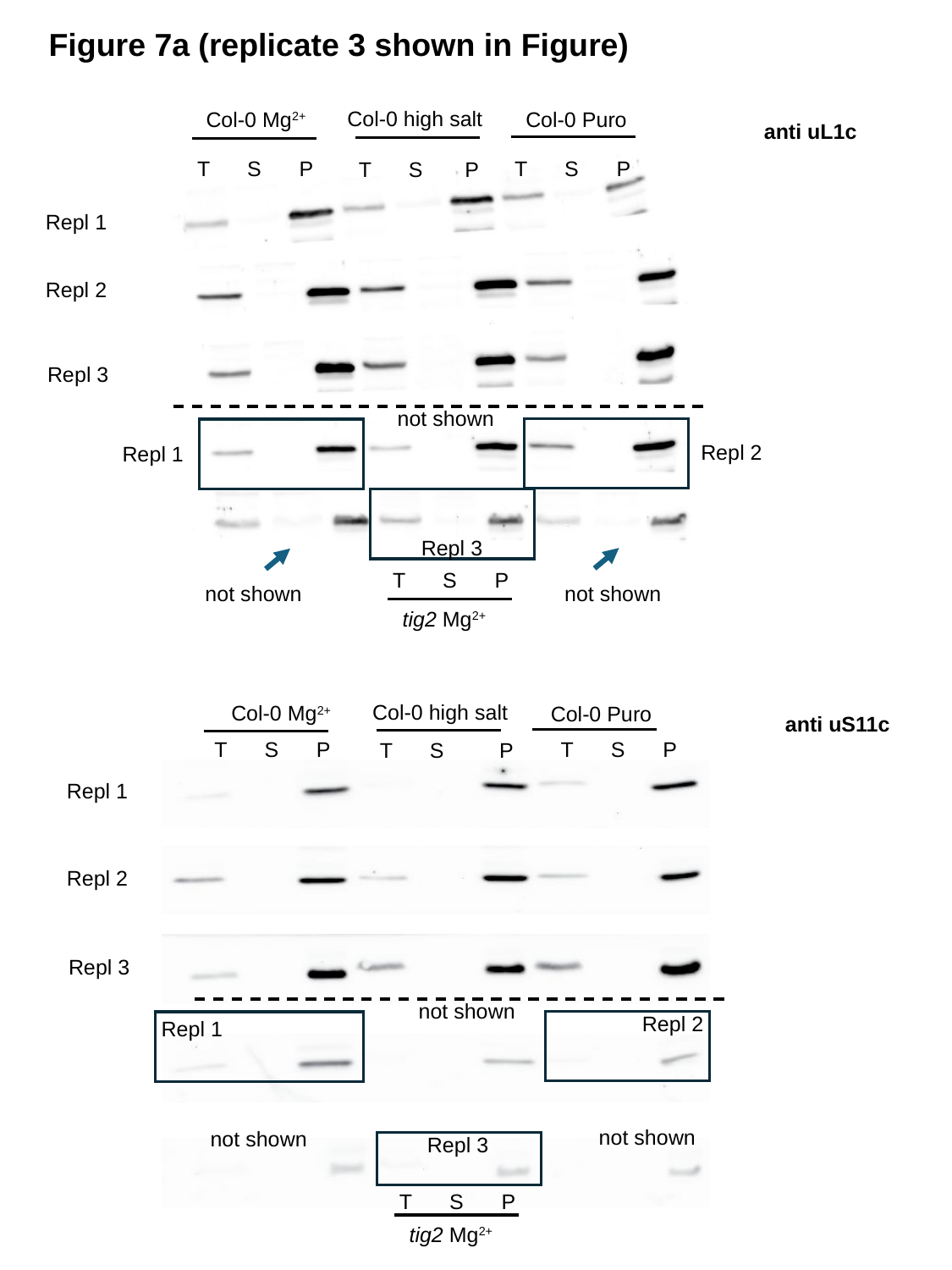

Figure 7a (replicate 3 shown in Figure)
Col-0 high salt
Col-0 Mg2+
Col-0 Puro
anti uL1c
T
S
P
T
S
P
T
S
P
Repl 1
Repl 2
Repl 3
not shown
Repl 2
Repl 1
Repl 3
T
S
P
not shown
not shown
tig2 Mg2+
Col-0 high salt
Col-0 Mg2+
Col-0 Puro
anti uS11c
T
S
P
T
S
P
T
S
P
Repl 1
Repl 2
Repl 3
not shown
Repl 2
Repl 1
not shown
not shown
Repl 3
T
S
P
tig2 Mg2+

## Slide 6
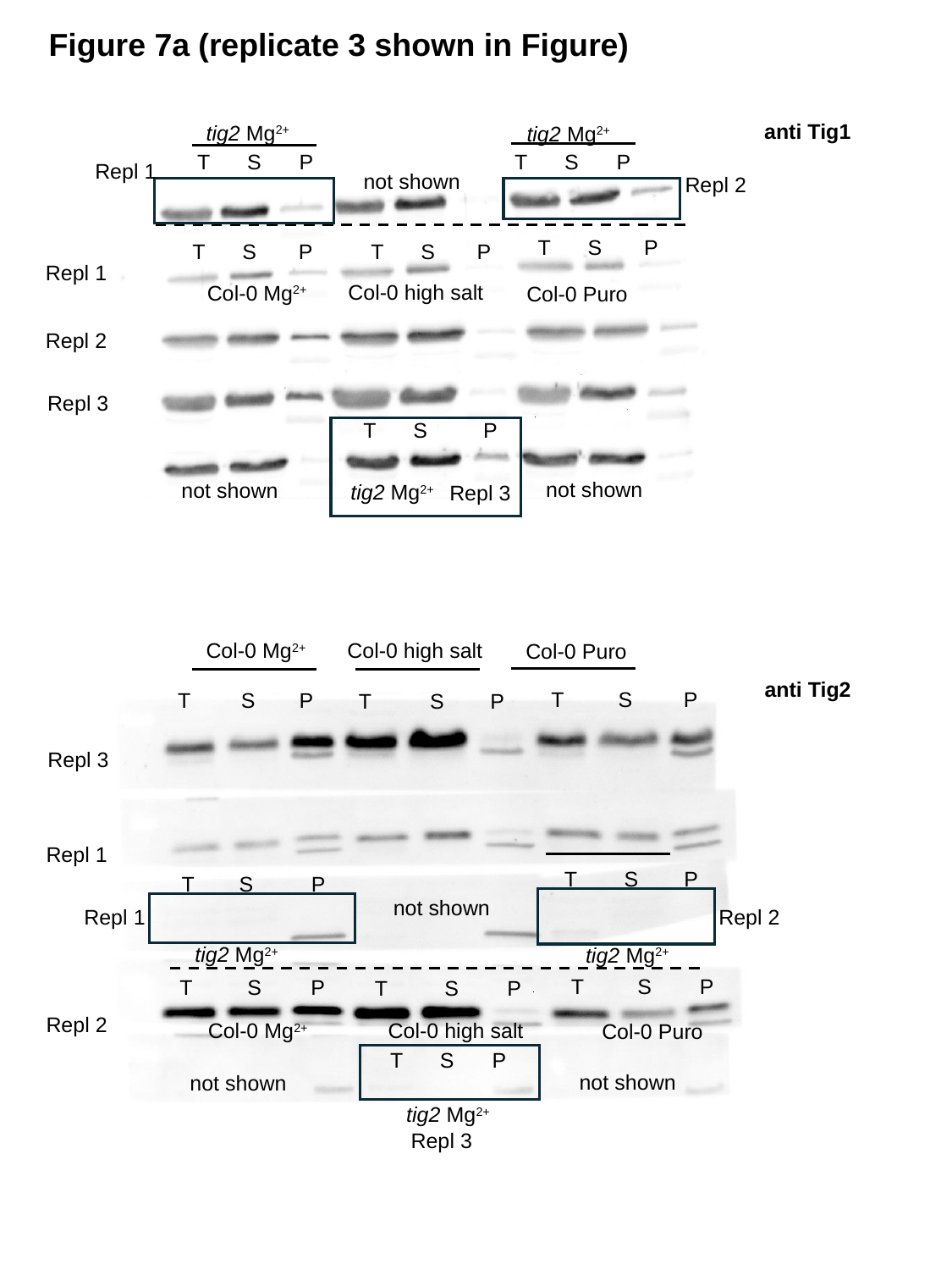

Figure 7a (replicate 3 shown in Figure)
anti Tig1
tig2 Mg2+
tig2 Mg2+
T
S
P
T
S
P
Repl 1
not shown
Repl 2
T
S
P
T
S
P
T
S
P
Repl 1
Col-0 high salt
Col-0 Mg2+
Col-0 Puro
Repl 2
Repl 3
T
S
P
not shown
not shown
tig2 Mg2+
Repl 3
Col-0 high salt
Col-0 Mg2+
Col-0 Puro
anti Tig2
T
S
P
T
S
P
T
S
P
Repl 3
Repl 1
T
S
P
T
S
P
not shown
Repl 1
Repl 2
tig2 Mg2+
tig2 Mg2+
T
S
P
T
S
P
T
S
P
Repl 2
Col-0 Mg2+
Col-0 high salt
Col-0 Puro
T
S
P
not shown
not shown
tig2 Mg2+
Repl 3

## Slide 7
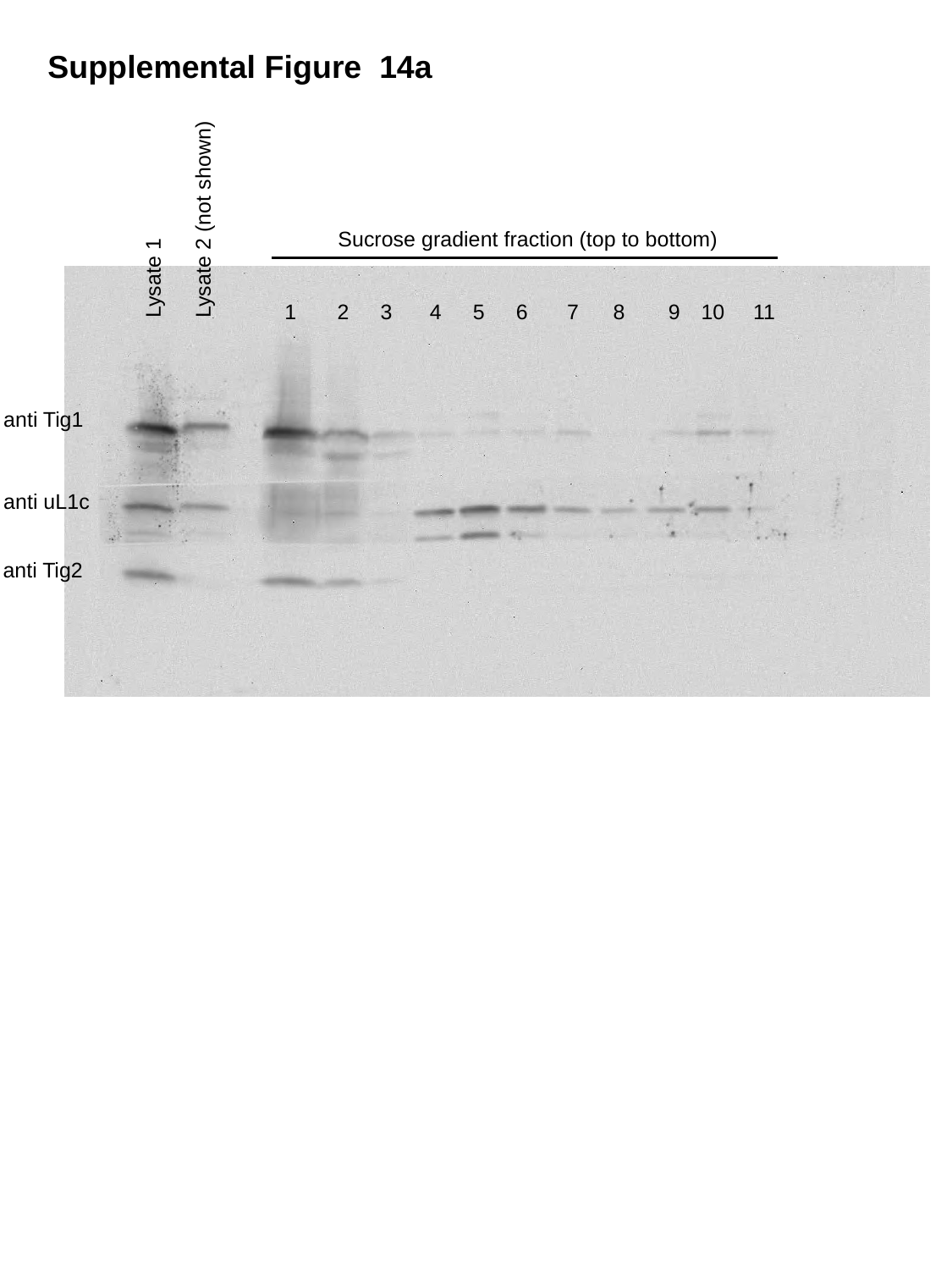

Supplemental Figure 14a
Lysate 2 (not shown)
Sucrose gradient fraction (top to bottom)
Lysate 1
1
2
3
4
5
6
7
8
9
10
11
anti Tig1
anti uL1c
anti Tig2
